# Synthesis of Water-Soluble Amino Functionalized Multithiacalix[4]arene via Quaternization of Tertiary Amino Groups

**DOI:** 10.3390/molecules23051117

**Published:** 2018-05-08

**Authors:** Roman Nosov, Pavel Padnya, Dmitriy Shurpik, Ivan Stoikov

**Affiliations:** A.M. Butlerov Chemistry Institute, Kazan Federal University, 420008 Kazan, Russia; romanosow@mail.ru (R.N.); padnya.ksu@gmail.com (P.P.); dnshurpik@mail.ru (D.S.)

**Keywords:** thiacalix[4]arene, receptor, aggregation, self-assembly, amino derivatives, nanoparticles, dendrimers, multithiacalix[4]arene

## Abstract

A convenient approach to the synthesis of multithiacalix[4]arene derivatives containing amino groups and phthalimide fragments by the formation of quaternary ammonium salts is presented. As the initial macrocycle for the synthesis of multithiacalix[4]arenes, a differently substituted *p-tert-*butylthiacalix[4]arene containing bromoacetamide and three phthalimide fragments was used in a *1,3-alternate* conformation. The macrocycle in *cone* conformation containing the tertiary amino groups was found to be a convenient core for the multithiacalix[4]arene systems. Interaction of the core multithiacalix[4]arene with monobromoacetamide derivatives of *p-tert-*butylthiacalix[4]arene resulted in formation in high yields of pentakisthiacalix[4]arene containing quaternary ammonium and phthalimide fragments. The removal of phthalimide groups led to the formation of amino multithiacalix[4]arene in a good yield. Based on dynamic light scattering, it was shown that the synthesized amino multithiacalix[4]arene, with pronounced hydrophobic and hydrophilic fragments, formed dendrimer-like nanoparticles in water via direct supramolecular self-assembly.

## 1. Introduction

Dendrimers are highly branched macromolecules with nanometer-scale dimensions and are defined by three components: a central core, an interior dendritic structure (branches), and an exterior surface with functional surface groups. The variable combination of these components provides products of different shapes and sizes with shielded interior cores that are ideal candidates for applications in both biological and materials science [1]. While the attached surface groups affect solubility and chelation ability, the variable cores create unique properties in terms of the cavity size, absorption capacity, and capture–release characteristics. A huge variety of dendrimers has been synthesized, with various core, branches, and surface functional groups [2,3,4,5,6]. Among all of the diverse classes of synthesized dendritic macromolecules, an interesting class is that based on various macrocyclic derivatives, e.g., crown ethers [7,8,9,10], cyclodextrins [11,12,13], pillar[n]arenes [14,15,16], resorcinarenes [17,18], and calix[n]arenes [19,20,21]. The ability to form inclusion complexes, the polyfunctionality of derivatives, and the ability to use spatial organization to create 3D nanoscale systems has attracted researchers to the creation of dendrimers based on macrocyclic derivatives. The macrocyclic platform of thiacalix[4]arene is one of the convenient building blocks for the creation of dendrimers [22]. The tendency to form inclusion compounds [23], the availability of mono-, di-, tri- and tetra-substituted derivatives [24,25,26], as well as the existence of spatially pre-organized conformational isomers [27] (*cone*, *partial cone*, *1,2-* and *1,3-alternate*) make the thiacalix[4]arene platform attractive for the preparation of polyfunctional dendrimers with well-oriented spatial organization. The literature presents a limited number of publications on the synthesis of multi(thia)calix[4]arene systems [28,29]. Previous studies have used a wide variety of reactions for the preparation of multi(thia)calix[4]arene systems, e.g., *O*-alkylation [30], hydrosilylation [31], amide formation [32,33], and “click” reactions [28,34]. However, there are almost no convenient approaches to the synthesis of multi(thia)calix[4]arenes with different functional groups on the surface in existing examples of multi(thia)calix[4]arene systems. This sharply limits the potential application of multi(thia)calix[4]arene systems. Moreover, the derivatives of multithiacalix[4]arenes able to dissolve in water are scarce. Earlier, it was shown in our group that water-soluble quaternary ammonium salts can be synthesized under mild conditions and in a high yield based upon the alkylation of *p-tert*-butylthiacalix[4]arene derivatives containing tertiary amine groups with alkyl halides [35]. Therefore, we decided to use this approach for the production of water-soluble multithiacalix[4]arenes using active bromo derivatives of *p-tert-*butylthiacalix[4]arene as alkylating agents. Primary amino groups were selected as surface functional groups to promote the dissolution of dendrimer-like structures in water and still permit the possibility of their further modification.

## 2. Results and Discussion

**Figure 1:**
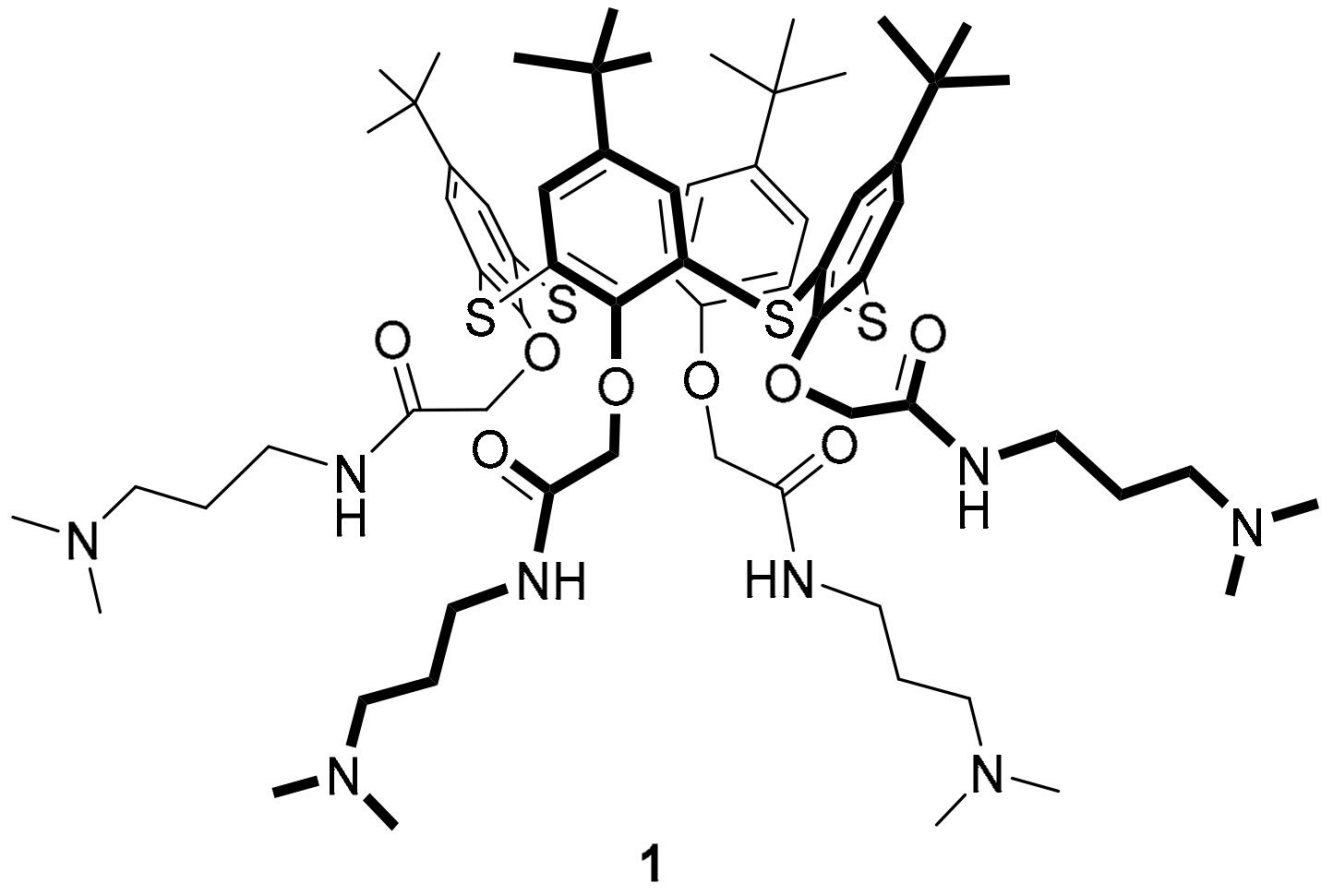
Core of multithiacalix[4]arene **1**.

As the core of the multithiacalix[4]arenes, we selected previously synthesized tetrasubstituted *p-tert-*butylthiacalix[4]arene **1** in the *cone* configuration (Figure 1). It contains polar tertiary amino groups on one side of the macrocycle and lipophilic *tert*-butyl groups on the opposite side [35]. The choice of this *p-tert-*butylthiacalix[4]arene as a core is primarily due to its synthetic availability, as well as the presence of reactive tertiary amine groups on sufficiently long spacers, which exclude the influence of the steric factors on the formation of products of incomplete alkylation. As a peripheral macrocycle, we decided to synthesize a differently substituted *p-tert-*butylthiacalix[4]arene in a *1,3-alternate* conformation containing phthalimide groups in addition to the bromoacetamide moiety. Phthalimide groups are convenient synthons of amino groups that can be easily converted into the corresponding primary amines in the presence of hydrazine hydrate or under conditions of acid catalysis. In addition, a less steric loading of the reaction center of the *p-tert-*butylthiacalix[4]arene derivative in the *1,3-alternate* conformation reduces the possibility of the formation of byproducts through partial alkylation of the core.

**Scheme 1:**
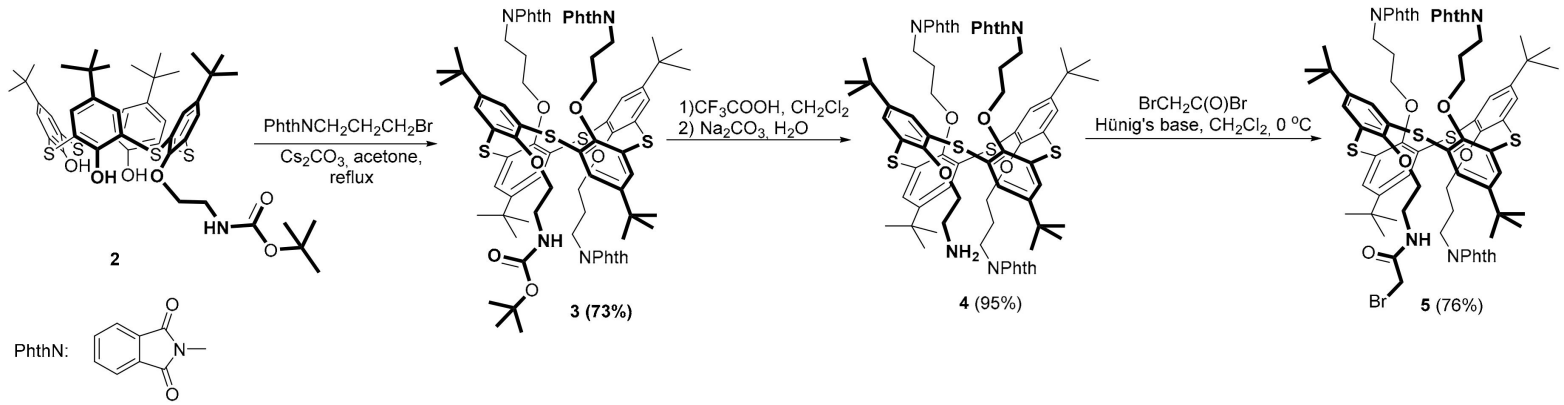
Synthesis of tetrasubstituted derivatives of thiacalix[4]arene **3**, **4** and **5**, from the monosubstituted thiacalix[4]arene **2**.

As the initial macrocycle for the synthesis of the precursor of multithiacalix[4]arenes, we chose mono-substituted *p-tert-*butylthiacalix[4]arene **2** [36] containing a *tert*-butyloxycarbonyl (BOC) protected aminoethoxy group. The presence of a BOC-protected amino group in macrocycle **2** will yield a differently substituted macrocycle containing phthalimide fragments and an amine group required to introduce a bromoacetamide moiety into the macrocycle structure. To introduce phthalimide fragments into the structure of the thiacalix[4]arene **2**, the interaction of macrocycle **2** with *N*-(3-bromopropyl)phthalimide in acetone was studied (Scheme 1). It is known that, depending on the metal carbonate (Na, K, or C), tetrafunctional derivatives of *p-tert*-butylthiacalix[4]arenes in the *cone*, *partial cone* and *1,3-alternate* conformations can be obtained [27]. Therefore, in order to obtain the tetrasubstituted derivative of *p-tert*-butylthiacalix[4]arene in the *1,3-alternate* configuration, cesium carbonate was chosen as the base in the reaction of macrocycle **2** with *N*-(3-bromopropyl)phthalimide. As a result of the reaction, macrocycle **3** was obtained with a yield of 73%. Based on ^1^H-^1^H Nuclear Overhauser Effect Spectroscopy (NOESY) NMR experiment (see Supporting Materials), macrocycle **3** is in a *1,3-alternate* conformation. 

Removal of the BOC group from the aminoethoxyl fragment of the thiacalix[4]arene **3** was a further step of our work. The synthesis was carried out in dichloromethane at room temperature in the presence of trifluoroacetic acid. As a result of the reaction, the monoamine **4** was synthesized in a high yield after neutralization with aqueous sodium carbonate. To introduce the bromoacetamide moiety into the structure of macrocycle **4**, the interaction of the monoamine **4** with bromoacetyl bromide in the presence of Hünig’s base (*N*,*N*-diisopropylethylamine) was studied. Macrocycle **5** was obtained with a yield of 76%. Based on the ^1^H-^1^H NOESY NMR spectra (see Supplementary Materials), macrocycle **5** had a *1,3-alternate* conformation. According to thin-layer chromatography (TLC), no other products were formed in the reaction. 

**Scheme 2:**
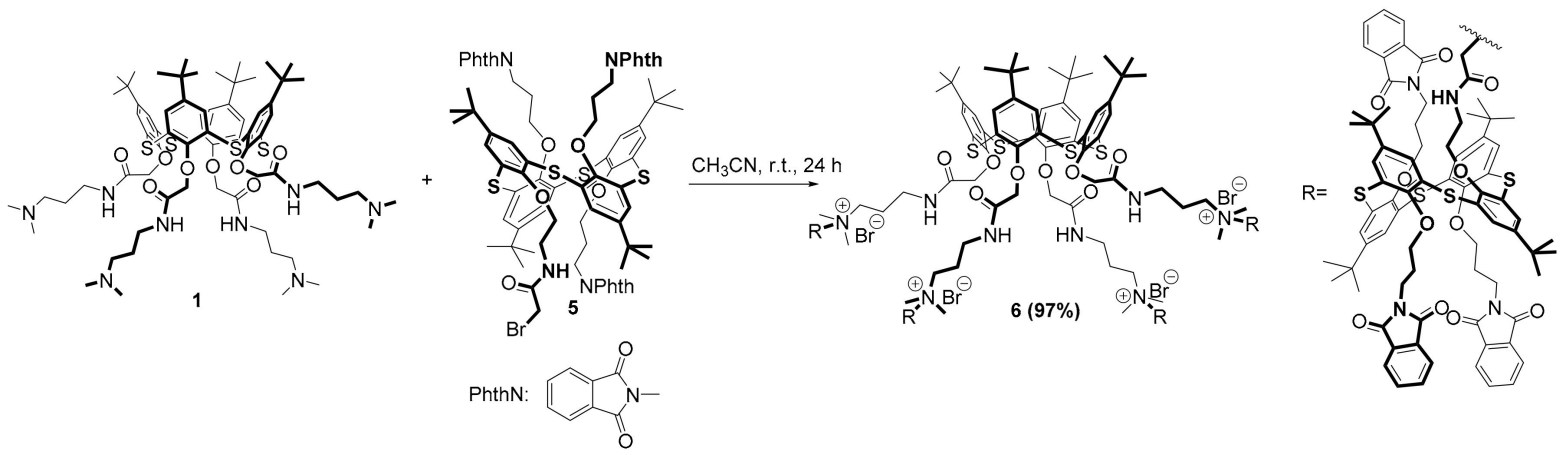
Alkylation of the tetrasubstituted macrocycle **1** with a monobromo derivative of thiacalix[4]arene **5** to generate multithiacalix[4]arene **6**.

Multithiacalix[4]arene containing phthalimide groups was obtained in the next step of the work. The interaction of macrocycle **1** with the thiacalix[4]arene **5** was carried out over 24 h (Scheme 2). The synthesis was carried out at room temperature in acetonitrile in order to reduce the possibility of the side reaction of the decomposition of quaternary ammonium derivatives. The advantage of using acetonitrile as a solvent was that the multithiacalix[4]arene **6** was poorly soluble and precipitated during the reaction. For the greatest degree of conversion of the tertiary amino groups, a 20% excess of macrocycle **5** was used against each functional group of the core **1**. It should be noted that a complex mixture of alkylation products of tertiary amino groups and of the products of the cleavage of quaternary ammonium salts by the Hoffmann reaction was formed when the reaction temperature was raised to 60 °C.

**Figure 2:**
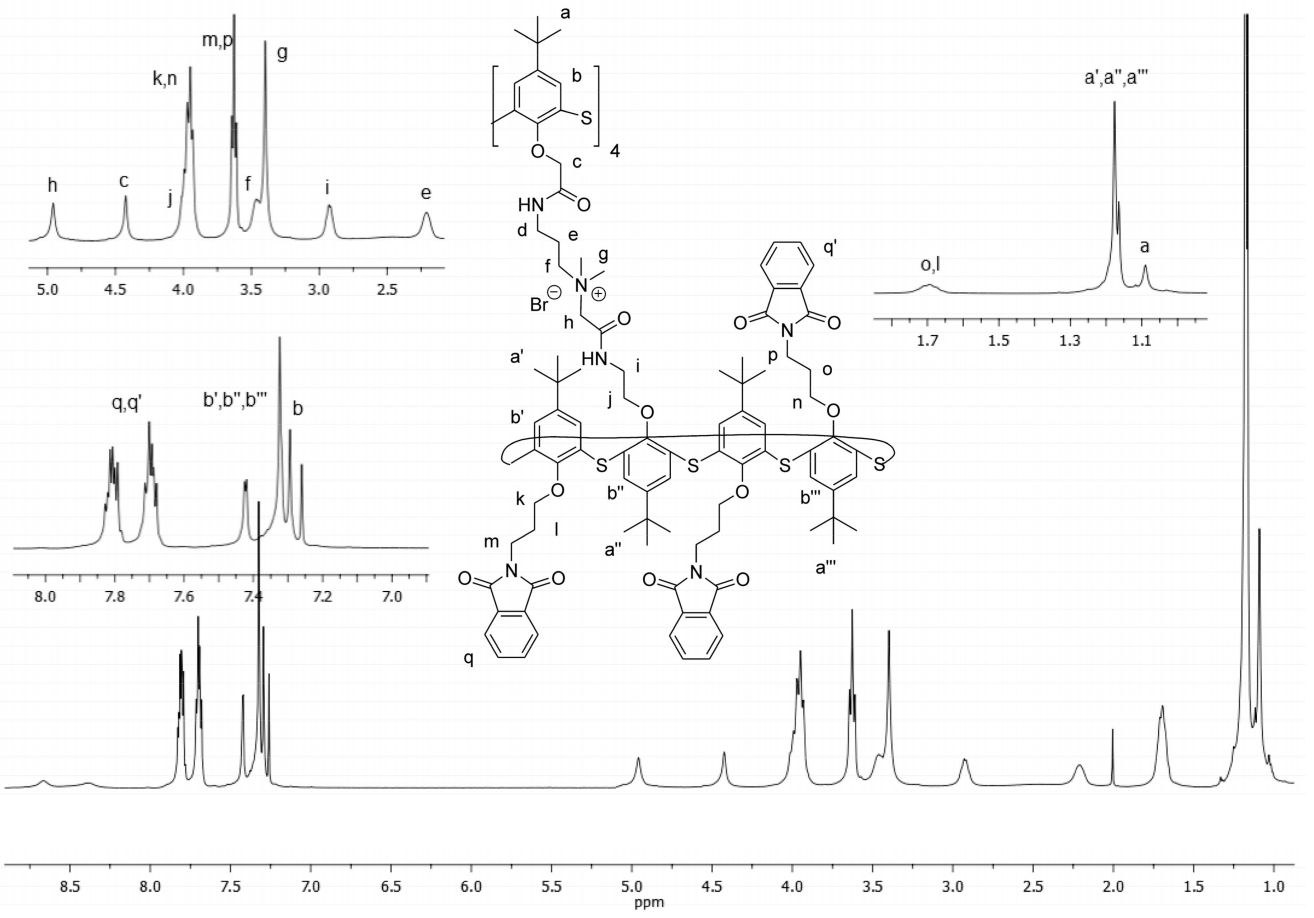
^1^H NMR spectrum of multithiacalix[4]arene **6**.

Multithiacalix[4]arene **6** was synthesized in a high yield, and its structure was characterized by NMR ^1^Н, ^13^С, and IR spectroscopy and Matrix-Assisted Laser Desorption/Ionization Time-of-Flight (MALDI-TOF) mass spectrometry. Figure 2 shows the NMR ^1^H spectrum of the synthesized multimacrocycle **6**. 

In the ^1^H NMR spectrum of macrocycle 6, individual assignments of the proton signals were made based on integrated intensity, multiplicity, chemical shifts, and comparison of spectral data of macrocycles **1** and **5** (see Supplementary Materials). A shift of signals to lower fields of methyl protons *g* (CH_3_-) 3.39 ppm, oxymethylene protons of the core *c* (-O-CH_2_-C (O)) 4.44 ppm and peripheral macrocycle *h* (-O-CH_2_-N^+^) 4.97 ppm against those observed in macrocycles **1** and **5** unequivocally indicates that the tertiary amino groups of macrocycle **1** were alkylated. Similar multiplicity of the signals in the ^1^H NMR spectrum of macrocycle **6** compared with those in macrocycles **1** and **5**, indicates the symmetrical structure of the multithiacalix[4]arene **6**. 

**Scheme 3:**
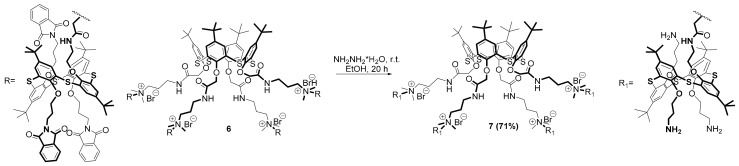
Hydrazinolysis of multithiacalix[4]arene **6** to generate of amino multithiacalix[4]arene **7**.

To obtain amino derivatives of the multithiacalix[4]arene, the hydrazinolysis of the multithiacalix[4]arene **6** (Scheme 3) was studied. It is known that quaternary ammonium derivatives can undergo various transformations in the presence of bases. Thus, removal of the phthalimide groups from multithiacalix[4]arene **6** was carried out at room temperature. Control over the conversion of phthalimide groups was performed by disappearance of the proton signals of the phthalimide groups in the ^1^H NMR spectra. 

**Figure 3:**
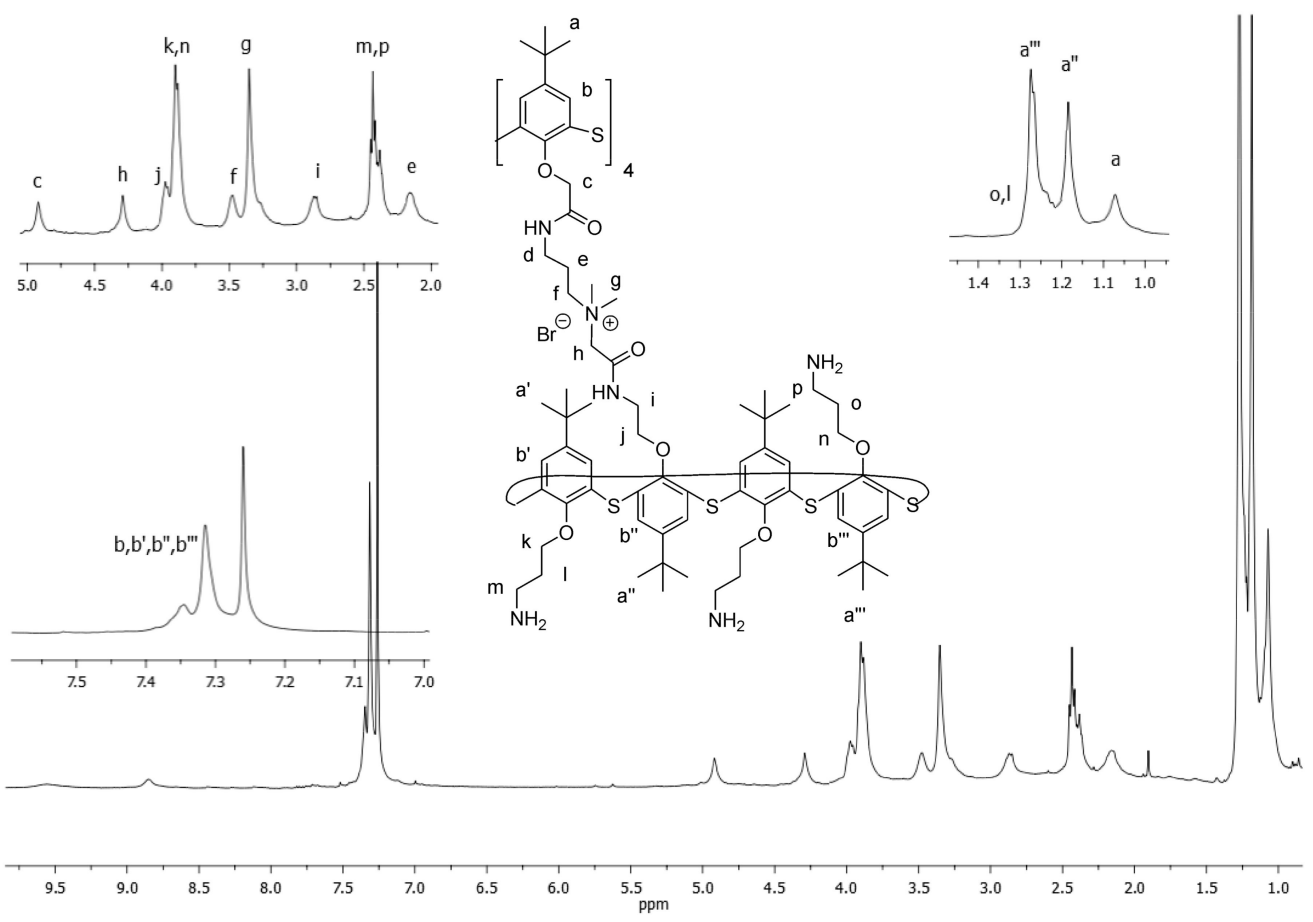
^1^H NMR spectrum of multithiacalix[4]arene **7**.

Multithiacalix[4]arene **7** was synthesized at a high yield, and its structure was characterized by NMR ^1^Н, ^13^С, IR spectroscopy, and MALDI-TOF mass spectrometry. Figure 3 shows the ^1^H NMR spectrum of the synthesized macrocycle **7**.

The disappearance of signals in the 7.5–8.0 ppm range clearly indicates complete conversion of the phthalimide groups to primary amino groups. In addition, the signals of the methylene protons *l, m* and *o, p* significantly shifted to strong fields. This can be explained by a change in their environment. In comparison with the chemical shifts of the methylene protons *o*, *l* (CH_2_-CH_2_-CH_2_-NPhth) 1.69 ppm and *m*, *p* (CH_2_-CH_2_-CH_2_-NPhth) 3.62 ppm of macrocycle **6**, in multimacrocycle **7** the signals of these groups are located in a strong field at 1.28 ppm and 2.45 ppm, respectively. It should also be noted that the absence of the proton signals of the alkene derivatives in the range of 5.5–6.5 ppm, as well as the symmetry of the signals in the ^1^H NMR spectrum of the multithiacalix[4]arene **7**, indicate that no byproducts of the cleavage of the multithiacalix[4]arene structure are formed during the hydrazinolysis. The appearance of a deformation vibration band of the NH bond of the primary amino group in the 1576 cm^−1^ region, as well as an increase in the intensity of the broad bands in the 3260–3360 cm^−1^ region vs. that in the spectrum of macrocycle **6** (see the Supplementary Materials), are further confirmation of the formation of amino groups in macrocycle **7**. Furthermore, the removal of the phthalimide groups led to a decrease in the absorption band of stretching vibrations of the carbonyl group (C=O)) in the region of 1683 cm^−1^.

**Figure 4:**
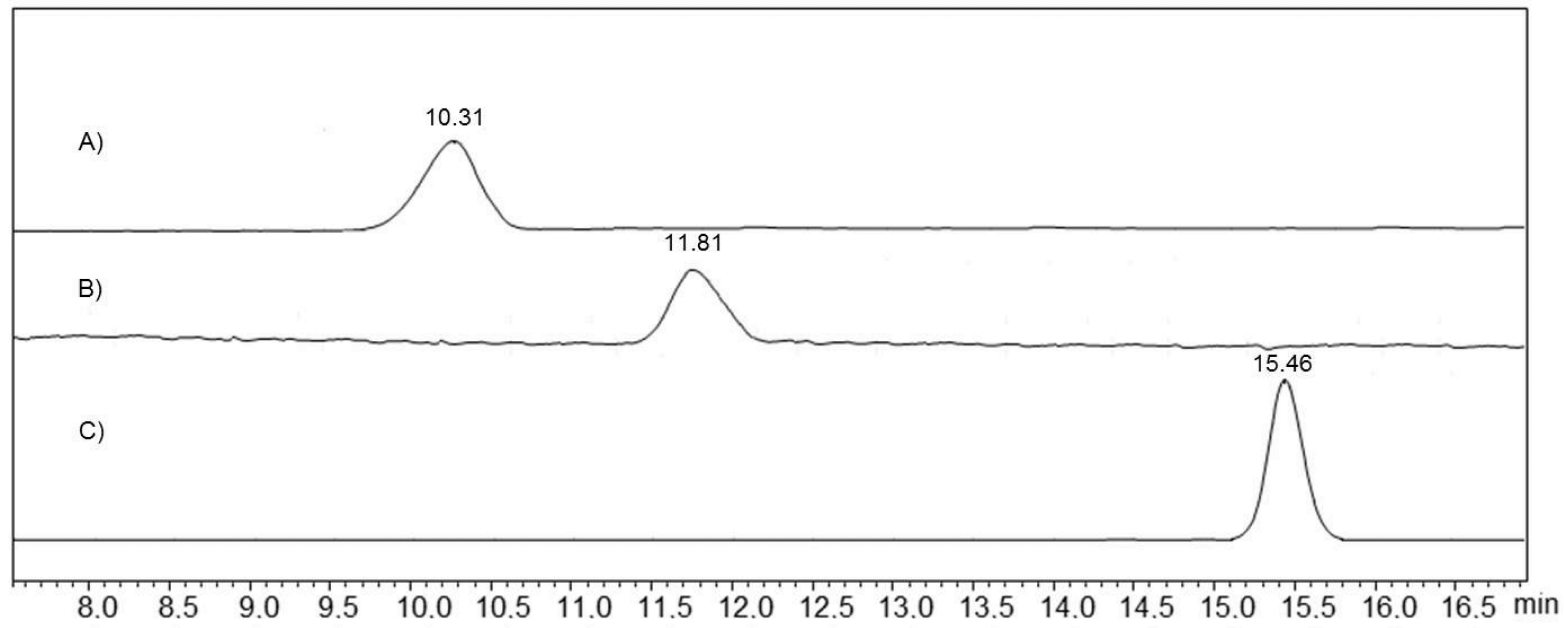
Gel permeation chromatography (GPC) chromatograms of multicalix[4]arenes **6**,**7** and macrocycle **5** (**A**) multithiacalix[4]arene **6**; (**B**) multithiacalix[4]arene **7**; (**C**) thiacalix[4]arene **5**.

Byproducts of the non-complete alkylation of core **1** in the synthesized multithiacalix[4]arene were estimated by the gel permeation chromatography (Figure 4). As follows from the data obtained, the synthesized multithiacalix[4]arenes **6** and **7** are represented by one peak at 10.31 min and 11.81 min, respectively, and the retention time of macrocycle **5** is equal to 15.46 min. The absence of extra peaks on the chromatograms of macrocycles **6** and **7** indicates that the multithiacalix[4]arenes obtained are free from the products of non-complete alkylation or cleavage of the quaternary ammonium salts.

**Figure 5:**
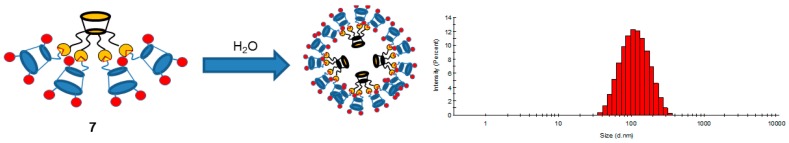
Dynamic light scattering (DLS) experiments: average size of aggregates of the multithiacalix[4]arene **7** ( C = 1 × 10^−5^ mol/L, d = 108.9 ± 3.2, pdi = 0.266).

The solubility in water and the possibility of their self-assembly with the formation of various nanosized particles is one of the important aspects of the use of dendrimers for various biomedical purposes. To study the behavior of amino multithiacalix[4]arene **7** in water, dynamic light scattering (DLS) was used. The macrocycle concentration varied in the range from 1 × 10^−3^ to 1 × 10^−5^ mol/L. DLS experiments showed that macrocycle **7** formed nanosized aggregates in water (108.9 ± 3.2 nm, pdi = 0.266) with a low polydispersity index at a macrocycle concentration of 1 × 10^−5^ mol/L (Figure 5).

With increasing macrocycle concentrations, colloidal systems are formed with a multimodal particle size distribution. Thus, amphiphilic multithiacalix[4]arene **7** with distinct hydrophobic (*tert*-butyl groups and aryl fragments) and hydrophilic (primary amino groups) parts of the molecule forms in water dendrimer-like nanoparticles by direct supramolecular self-assembly. The obtained results offer new perspectives for the creation in water of the supramolecular nanostructures by directional self-assembly of pre-constructed building blocks. The next stage of our work will involve extension of the synthetic approach to water-soluble amino multithiacalix[4]arene systems with other macrocyclic and acyclic cores and modification of the amino groups of the synthesized multi-macrocycle by various functional groups. 

## 3. Materials and Methods

### 3.1. General Experimental Information

All reagents and solvents were used directly as purchased or purified according to the standard procedures. Analytical thin-layer chromatography was carried out using commercial silica gel plates and visualization was effected with short wavelength UV light (254 nm). Column chromatography was performed with silica gel 60 H, slurry packed. NMR spectra were recorded at 400 MHz for ^1^H, and 100 MHz for ^13^C with CDCl_3_ as solvent. Chemical shifts are reported in delta (δ) units in parts per million (ppm) and splitting patterns are designated as s, singlet; d, doublet; t, triplet; q, quartet; m, multiplet and br, broad. Coupling constants are recorded in Hertz (Hz). The structure of the products was determined by a combination of spectroscopic methods such as IR, 1D and 2D NMR NOESY experiment and MALDI-MS. IR spectra were recorded with Spectrum 400 IR spectrometer (Perkin Elmer, Waltham, MA, USA). Absorbance frequencies are expressed in reciprocal centimeters (cm^–1^). MALDI spectra were recorded using an Ultraflex III mass spectrometer (Bruker Daltonik GmbH, Bremen, Germany) with 4-nitroaniline as a matrix. Peaks of molecular ions are represented by the most abundant mass. Melting points were determined using the Melting Point Apparatus SMP10 (Cole-Parmer Ltd., Stone, Staffordshire, UK). First grade Millipore^®^ water was prepared from distilled water on Simplicity 185 (Millipore S.A.S., Molsheim, France). GPC analyses were performed over a GPC column (Agilent PLGel 3 µm Mixed-E, 25 mm) using an Agilent 1200 Liquid Chromatograph (Agilent, Omaha, NE, USA) equipped with a refractometer. Macrocycles **1** and **2** were synthesized according to a procedure reported in the literature [35,36]. 

### 3.2. Synthesis of 5,11,17,23-Tetra-tert-butyl-25,26,27-tri[3′-(N-phthalimido)propoxy]-28-[tert-butyl(2′-aminoethoxy)carbamate]-2,8,14,20-tetrathiacalix[4]arene (1,3-alternate) ***3***

A mixture of 0.53 g (0.58 mmol) of the compound **2** and 1.50 g (4.60 mmol) of Cs_2_CO_3_ in 50 mL of acetone was heated under reflux for 30 min, after which 1.23 g (4.59 mmol) of *N*-(3-bromopropyl)phthalimide was added. The reaction mixture was heated under reflux for 18 h, after which the solvent was removed, and 20 mL of chloroform, 15 mL of distilled water was added. The mixture was stirred at room temperature for 30 min. The organic layer was separated, washed with water (3 × 15 mL), and dried over anhydrous Na_2_SO_4_. The solvent was removed at reduced pressure. Excess of *N*-(3-bromopropyl)phthalimide was removed by boiling the precipitate with hexane. Compound **3** was obtained from column chromatography (trichlormethane-ethanol 60:1). 

5,11,17,23-Tetra-*tert*-butyl-25,26,27-[3′-(*N*-phthalimido)propoxy]-28-[*tert*-butyl(2′-aminoethoxy)carbamate]-2,8,14,20-tetrathiacalix[4]arene (*1,3-alternate*) **3** was obtained as a white solid at a yield of 73% (0.61 g). Mp 194–195 °С. IR (KBr/cm^−1^): 1710 (C(O)-NH), 3440 (-NH); ^1^H-NMR, CDCl_3_-d1, δ (ppm): 1.20 (s, 9H, (CH_3_)_3_C), 1.22 (s, 27H, (CH_3_)_3_C), 1.43 (s, 9H, (CH_3_)_3_C-О), 1.77 (m, 6H, CH_2_-CH_2_-CH_2_), 2.98 (m, 2H, CH_2_-CH_2_-NH-), 2.98 (m, 2H, CH_2_-CH_2_-NH-), 3.62 (t, 2H, J = 7.0 Hz, -CH_2_-CH_2_-Phth,), 3.69 (t, 4H, J = 6.7 Hz -CH_2_-CH_2_-Phth,), 3.98 (m, 4H, -CH_2_-CH_2_-Phth), 4.02 (m, 2H, CH_2_-CH_2_-NH-), 5.51 (s, 1H, -NH), 7.36 (br.s, 2H, Ar-H), 7.37 (br.s., 2H, Ar-H), 7.39 (m, 2H, Ar-H), 7.41 (m, 2H, Ar-H), 7.73 (m, 6H, Ar’-Phth), 7.85 (m, 6H, Ar’-Phth). ^13^C-NMR, δ (ppm): 28.46, 28.79, 28.85, 30.98, 31.15, 31.21, 34.10, 34.14, 34.19, 35.19, 35.24, 67.67, 67.90, 69.91, 123.22, 127.74, 128.12, 128.30, 128.44, 129.49, 129.63, 130.00, 130.05, 132.07, 132.11, 133.88, 145.85, 145.92, 155.88, 157.03, 157.31, 157.50, 168.09, 168.12. MALDI–TOF MS (4-nitroaniline) analysis shows a signal at *m*/*z* = 1426.9 corresponding to [М + H]^+^ (calc. mass for M (C_80_H_88_N_4_O_12_S_4_): 1425.8) Elemental analysis for (C_80_H_88_N_4_O_12_S_4_). Calculated (%): C, 67.39, H, 6.22, N, 3.93, S, 8.99. Found (%): C, 67.77 %, H, 6.28%, N, 3.88%, S, 9.17%. 

### 3.3. Synthesis of 5,11,17,23-Tetra-tert-butyl-25,26,27-tri[3′-(N-phthalimido)propoxy]-28-[2′-aminoethoxy]-2,8,14,20-tetrathiacalix[4]arene (1,3-alternate) ***4***

In a round bottom flask of 100 mL 0.50 g (0.35 mmol) of macrocycle **3**, 1.43 mL (18.8 mmol) of trifluoroacetic acid, 20 mL of dichloromethane and 5 mL of water were added. The reaction mixture was stirred for 24 h at 30 °C. The solvent was evaporated, and the residue was dissolved in 20 mL chloroform. The organic layer was washed with 5% NaHCO_3_ and water (3 × 20 mL), diluted, and evaporated to dryness. 

5,11,17,23-Tetra-*tert*-butyl-25,26,27-[3′-(*N*-phthalimido)propoxy]-28-[2′-aminoethoxy]-2,8,14,20-tetrathiacalix[4]arene (*1,3-alternate*) **4** was obtained as a white solid at a yield of 95% (0.44 g). Mp 125–126 °С. IR (KBr/cm^−1^): 1707 (C(O)-NH); ^1^H-NMR, CDCl_3_-d1, δ (ppm): 1.21 (s, 9H, (CH_3_)_3_C), 1.23 (s, 27H, (CH_3_)_3_C), 1.61 (m, 4H, CH_2_-CH_2_-CH_2_), 1.7 1 (m, 2H, CH_2_-CH_2_-CH_2_), 2.39 (m, 2H, CH_2_-CH_2_-NH-), 3.62 (m, 6H, -CH_2_-CH_2_-Phth), 3.89 (m, 2H, CH_2_-CH_2_-NH-), 3.91-3.97 (m, 6H, -CH_2_-CH_2_- CH_2-_Phth), 7.31 (d, 2H, J = 2.4 Hz, Ar-H), 7.34 (d, 2H, J = 2.4 Hz, Ar-H), 7.34 (s., 2H, Ar-H), 7.37 (s, 2H, Ar-H), 7.68–7.84 (m, 12H, Ar’-Phth). ^13^C-NMR, δ (ppm): 168.78, 168.11, 157.14, 156.65, 154.41, 147.59, 146.97, 146.71, 134.46, 133.96, 133.83, 132.05, 131.53, 129.77, 129.70, 128.54, 128.34, 127.88, 127.79, 127.51, 126.67, 123.67, 123.27, 68.46, 67.39, 63.86, 39.69, 36.51, 35.13, 34.37, 34.26, 31.14, 31.03, 29.70, 29.07, 28.61, 28.23. MALDI–TOF MS (4-nitroaniline) analysis shows a signal at *m*/*z* = 1325.4 corresponding to [М + H]^+^ (calc. mass for M (C_75_H_80_N_4_O_10_S_4_): 1324.5) Elemental analysis for (C_75_H_80_N_4_O_10_S_4_). Calculated (%): C, 67.95, H, 6.08, N, 4.23, S, 9.67. Found (%): C, 68.14%, H, 6.34%, N, 4.57%, S, 10.15%.

### 3.4. Synthesis of 5,11,17,23-Tetra-tert-butyl-25,26,27-tri[3′-(N-phthalimido)propoxy]-28-[2′-bromoacetamidethoxy]-2,8,14,20-tetrathiacalix[4]arene (1,3-alternate) ***5***

In a round bottom flask of 100 mL equipped with a dropping funnel and ace bath, 0.50 g (0.38 mmol) of compound **4** and 0.26 mL (6.2 mmol) of *N*,*N*-diisopropylethylamine in 50 mL of dry dichloromethane were added. A solution of bromoacetyl bromide 0.083 g (0.42 mmol) in 10 mL of dry dichloromethane was placed in the dropping funnel and added to the reaction mixture. After dropping of bromoacetyl bromide solution, the reaction mixture was stirred for 2 h at room temperature, and water was added. The organic layer was separated and washed three times with 15 mL of water. Dichloromethane was evaporated and macrocycle **5** was obtained with column chromatography (eluent dichloromethane:methanol 100:1).

5,11,17,23-Tetra-*tert*-butyl-25,26,27-[3′-(*N*-phthalimido)propoxy]-28-[2′-bromoacetamidethoxy]-2,8,14,20-tetrathiacalix[4]arene (1,*3-alternate*) **5** was obtained as a slightly yellow solid at a yield of 76% (0.41 g). M.p. 180–181 °С. IR (KBr/cm^−1^): 1708 (C(O)-NH), 3384 (-NH); ^1^H-NMR, CDCl_3_-d1, δ (ppm): 1.16 (s, 18H, (CH_3_)_3_C), 1.19 (s, 9H, (CH_3_)_3_C), 1.20 (s, 9H, (CH_3_)_3_C), 1.26 (s, 9H, (CH_3_)_3_C), 1.75 (m, 6H, CH_2_ -CH_2_-CH_2_), 3.20 (q, 2H, J = 5.6 Hz, CH_2_-CH_2_-NH-), 3.54 (t, 2H, J = 7.3 Hz -CH_2_-CH_2_-Phth), 3.67 (t, 4H, J = 6.7 Hz,-CH_2_-CH_2_-Phth,), 3.79 (s, 2H, -CH_2_-NH-), 3.97 (m, 2H, O-CH_2_-CH_2_-CH_2_), 3.99 (m, 4H, O-CH_2_-CH_2_-CH_2_) 4.07 (m, 2H, CH_2_-CH_2_-NH-), 7.33 (d, 2H, J = 2.4 Hz, AB system Ar-H), 7.37 (br.s, 4H, Ar-H), 7.38 (br.s, 2H, Ar-H), 7.45 (br.t, 1H, NH), 7.68-7.78 (m, 12H, Ar’-H). ^13^C-NMR, δ (ppm): 188.13, 173.25, 168.14, 168.07, 165.60, 157.47, 157.38, 156.67, 145.93, 145.89, 145.63, 133.95, 133.91, 132.09, 132.02, 129.95, 129.79, 129.57, 129.38, 128.50, 128.23, 127.92, 127.35, 123.24, 69.20, 67.91, 67.72, 40.10, 35.54, 35.17, 34.46, 34.18, 32.24, 32.19, 31.13, 30.18, 29.72, 29.01, 28.88, 28.84. MALDI–TOF MS (4-nitroaniline) analysis shows a signal at *m*/*z* = 1469.7 corresponding to [М + Na]^+^ and *m*/*z*(av.) = 1485.7 corresponding to [М + K]^+^ (calc. mass for M(av.) (C_77_H_81_BrN_4_O_11_S_4_): 1446.6) Elemental analysis for (C_77_H_81_BrN_4_O_11_S_4_). Calculated (%): C, 63.93; H, 5.64; N, 3.87; S, 8.86. Found (%):C, 64.08, H, 5.71, N, 3.98, S, 9.01. 

### 3.5. Synthesis of Multithiacalix[4]arene ***6***

In a round-bottomed flask, 0.50 g (0.35 mmol) of macrocycle **1**, 0.11 g (0.088 mmol) of macrocycle **5** and 40 mL of acetonitrile were added. The reaction mixture was stirred for 24 h at 25 °C. The precipitate was filtered off and washed with acetonitrile. The residue was dried in a vacuum desiccator. 

Multithiacalix[4]arene **6** was obtained as a slightly yellow solid at a yield of 97% (0.60 g). M.p. 154–155 °С. IR (KBr/cm^−1^): 1709 (C(O)-NH), 3204 (-NH); ^1^H-NMR, CDCl_3_-d1, δ (ppm): 1.09 (s, 36H, (CH_3_)_3_C), 1.16 (s, 36H, (CH_3_)_3_C), 1.18 (s, 108H, (CH_3_)_3_C), 1.69 (m, 24H, CH_2_-CH_2_-CH_2_), 3.39 (br.s., 24H, CH_3_-N-), 3.46 (m, 8H, -NH-CH_2_-CH_2_-CH_2_-N^+^), 3.62(m, 24H, -CH_2_-CH_2_-Phth,), 3.94 (m, 24H, O-CH_2_-CH_2_-CH_2_-Phth-), 3.97 (m, 8H, NH-CH_2_-CH_2_-CH_2_-N^+^), 3.99 (m, 8H, O-CH_2_-CH_2_-NH-), 4.42 (s, 8H, O-CH_2_-C(O)-NH-), 4.95 (s, 8H, -NH-CH_2_-N^+^), 7.29 (br.s., 8H, Ar-H core), 7.27–7.48 (m, 32H, Ar-H), 7.64–7.89 (m, 48H, Ar-H Phth), 8.37 (br.s, 4H, NH), 8.67 (br.s, 4H, NH). ^13^C-NMR, δ (ppm): 169.12, 168.26, 168.21, 162.81, 157.36, 156.34, 147.83, 146.22, 146.06, 145.90, 135.00, 134.15, 133.98, 132.21, 132.14, 129.32, 129.17, 128.64, 128.43, 128.40, 128.31, 128.20, 123.37, 123.32, 74.50, 67.68, 65.39, 64.87, 62.96, 51.88, 38.79, 36.38, 35.38, 35.26, 34.43, 34.38, 34.29, 34.25, 31.42, 31.42, 31.26, 31.26, 31.22, 31.22, 28.95, 28.91, 23.25. MALDI–TOF MS (4-nitroaniline) analysis shows a signal at *m*/*z* = 7074.2 corresponding to [М + H]^+^ (calc. mass for M(C_376_H_428_Br_4_N_24_O_52_S_20_): 7073.3) Elemental analysis for (C_376_H_428_Br_4_N_24_O_52_S_20_). Calculated (%): C, 63.75; H, 6.20; N, 4.75; S, 9.05. Found (%): C, 63.98, H, 6.35, N, 4.91, S, 9.17. 

### 3.6. Synthesis of Multithiacalix[4]arene ***7***

In a 100-mL round-bottomed flask, 0.50 g (0.071 mmol) of multithiacalix[4]arene **6**, 0.69 mL (14.2 mmol) of hydrazine hydrate, 20 mL of ethanol and 20 mL of THF were added. The reaction mixture was stirred for 24 h at 25 °C. The solvent was evaporated, and the residue was dissolved in 20 mL chloroform. The organic layer was washed with water (3 × 20 mL), diluted, and evaporated to dryness. 

Multithiacalix[4]arene **7** was obtained as a slightly yellow solid at a yield of 71% (0.28 g). M.p. 141–142 °С. IR (KBr/cm^−1^): 1683, 1709 (C(O)-NH), 3350 (-NH); ^1^H-NMR, CDCl_3_-d1, δ (ppm): 1.07 (s, 36H, (CH_3_)_3_C), 1.18 (s, 76H, (CH_3_)_3_C), 1.18–1.27 (m, 24H, O-CH_2_-CH_2_-CH_2_-Phth-), 1.27 (s, 76H, (CH_3_)_3_C), 2.17 (m, 8H, -NH-CH_2_-CH_2_-CH_2_-N^+^), 2.38–2.43 (m, 24H, -O-CH_2_-CH_2_-CH_2_-Phth), 2.87 (m, 8H, -O-CH_2_-CH_2_-NH), 3.35 (br.s., 24H, -N^+^-(CH_3_)_2_), 3.43 (br.s., 8H, NH-CH_2_-CH_2_-CH_2_-N^+^), 3.90 (m, 24H, -CH_2_-CH_2_-Phth), 3.97 (m, 8H, O-CH_2_-CH_2_-NH-), 4.29 (br.s, 8H, O-CH_2_-C(O)-NH-), 4.92 (br.s, 8H, -NH-CH_2_-N^+^), 7.31 (m., 32H, Ar-H), 7.35 (br.s., 8H, Ar-H core), 8.84 (br.s, 4H, NH), 9.57 (br.s, 4H, NH). ^13^C-NMR, δ (ppm): 157.36, 146.43, 146.10, 128.80, 128.57, 128.50, 128.31, 128.02, 127.43, 127.24, 67.65, 51.43, 51.18, 38.81, 38.61, 34.35, 32.74, 32.10, 31.26, 29.71, 23.18. MALDI–TOF MS (4-nitroaniline) analysis shows a signal at *m*/*z* = 5514.2 corresponding to [М + H]^+^ (calc. mass for M (C_280_H_404_Br_4_N_24_O_28_S_20_): 5512.3) Elemental analysis for (C_280_H_404_Br_4_N_24_O_28_S_20_). Calculated (%):C, 60.89; H, 7.52; N, 6.09; S, 11.61 Found (%): C, 61.12; H, 7.64; N, 6.17; S, 11.76.

## 4. Conclusions

An easy synthetic approach is shown to be able to obtain water-soluble amino functionalized multithiacalix[4]arene derivatives with primary and quaternary amino groups and phthalimide fragments. The structures of macrocycles and multithiacalixarenes were characterized using IR as well as one-dimensional and two-dimensional NMR spectroscopy. Dynamic light scattering showed that multithiacalix[4]arene with distinct hydrophobic (*tert*-butyl groups and aryl fragments) and hydrophilic (primary amino groups) parts of the molecule formed dendrimer-like nanoparticles in water by directional supramolecular self-assembly. The results offer new prospects for the creation of supramolecular nanostructures in aqueous media. The developed synthetic approach can be further extended to macrocyclic and acyclic cores, and further functionalization of the synthesized amino multithiacalix[4]arene makes it possible to vary both its complexing properties and the hydrophilic–lipophilic balance of the compounds.

## Supplementary Materials

The following are available online, Figure S1: NMR ^1^H spectrum of macrocycle **1**. Figure S2: IR spectrum of macrocycle **1**. Figure S3: NMR ^1^H spectrum of compound **3**. Figure S4: NMR ^13^C spectrum of compound **3**. Figure S5: NMR ^1^H-^1^H NOESY spectrum of compound **3**. Figure S6: MALDI TOF spectrum of compound **3**. Figure S7: NMR ^1^H spectrum of macrocycle **4**. Figure S8: NMR ^13^С spectrum of macrocycle **4**. Figure S9: MALDI-TOF mass spectrum of macrocycle **4**. Figure S10: NMR ^1^H spectrum compound **5**. Figure S11: NMR ^13^C spectrum of compound **5**. Figure S12: NMR ^1^H-^1^H NOESY spectrum of compound **5**. Figure S13: MALDI TOF mass spectrum of compound **5**. Figure S14: IR spectrum of compound **5**. Figure S15: NMR ^1^H spectrum of multithiacalix[4]arene **6**. Figure S16: NMR ^13^C spectrum of multithiacalix[4]arene **6**. Figure S17: MALDI TOF mass spectrum of multithiacalix[4]arene **6**. Figure S18: IR spectrum of multithiacalix[4]arene **6**. Figure S19: NMR ^1^H spectrum of multithiacalix[4]arene **7**. Figure S20: NMR ^13^C spectrum of multithiacalix[4]arene **7**. Figure S21: IR spectrum of multithiacalix[4]arene **7**. Figure S22: MALDI TOF mass spectrum of multithiacalix[4]arene **7**.
