# Supplementary material for: Synthesis of Water-Soluble Amino Functionalized Multithiacalix[4]arene via Quaternization of Tertiary Amino Groups
Source: Molecules. 2018 May 8;23(5):1117. doi: 10.3390/molecules23051117 (PMC6100039; doi:10.3390/molecules23051117)

Synthesis of Water-Soluble Amino Functionalized Multithiacalix[4]arene via Quaternization of Tertiary Amino Groups

Roman Nosov, Pavel Padnya, Dmitriy Shurpik and Ivan Stoikov*

Kazan Federal University, Kremlevskaya, 18, Kazan, Russian Federation; public.mail@kpfu.ru

Table of Contents:

Figure 1S. NMR ^1H spectrum of macrocycle 1

Figure 2S. IR spectrum of macrocycle 1

Figure 3S. NMR ^1H spectrum of compound 3

Figure 4S. NMR ^{13}C spectrum of compound 3

Figure 5S. NMR ^1H-^1H NOESY spectrum of compound 3

Figure 6S. MALDI TOF spectrum of compound 3

Figure 7S. NMR ^1H spectrum of macrocycle 4

Figure 8S. NMR ^{13}C spectrum of macrocycle 4

Figure 9S. MALDI-TOF mass spectrum of macrocycle 4

Figure 10S. NMR ^1H spectrum compound 5

Figure 11S. NMR ^{13}C spectrum of compound 5

Figure 12S. NMR ^1H-^1H NOESY spectrum of compound 5

Figure 13S. MALDI TOF mass spectrum of compound 5

Figure 14S. IR spectrum of compound 5

Figure 15S. NMR ^1H spectrum of multithiacalix[4]arene 6

Figure 16S. NMR ^{13}C spectrum of multithiacalix[4]arene 6

Figure 17S. MALDI TOF mass spectrum of multithiacalix[4]arene 6

Figure 18S. IR spectrum of multithiacalix[4]arene 6

Figure 19S. NMR ^1H spectrum of multithiacalix[4]arene 7

Figure 20S. NMR ^{13}C spectrum of multithiacalix[4]arene 7

Figure 21S. IR spectrum of multithiacalix[4]arene 7

Figure 22S. MALDI TOF mass spectrum of multithiacalix[4]arene 7

Figure 1S. NMR ^1H spectrum of macrocycle 1

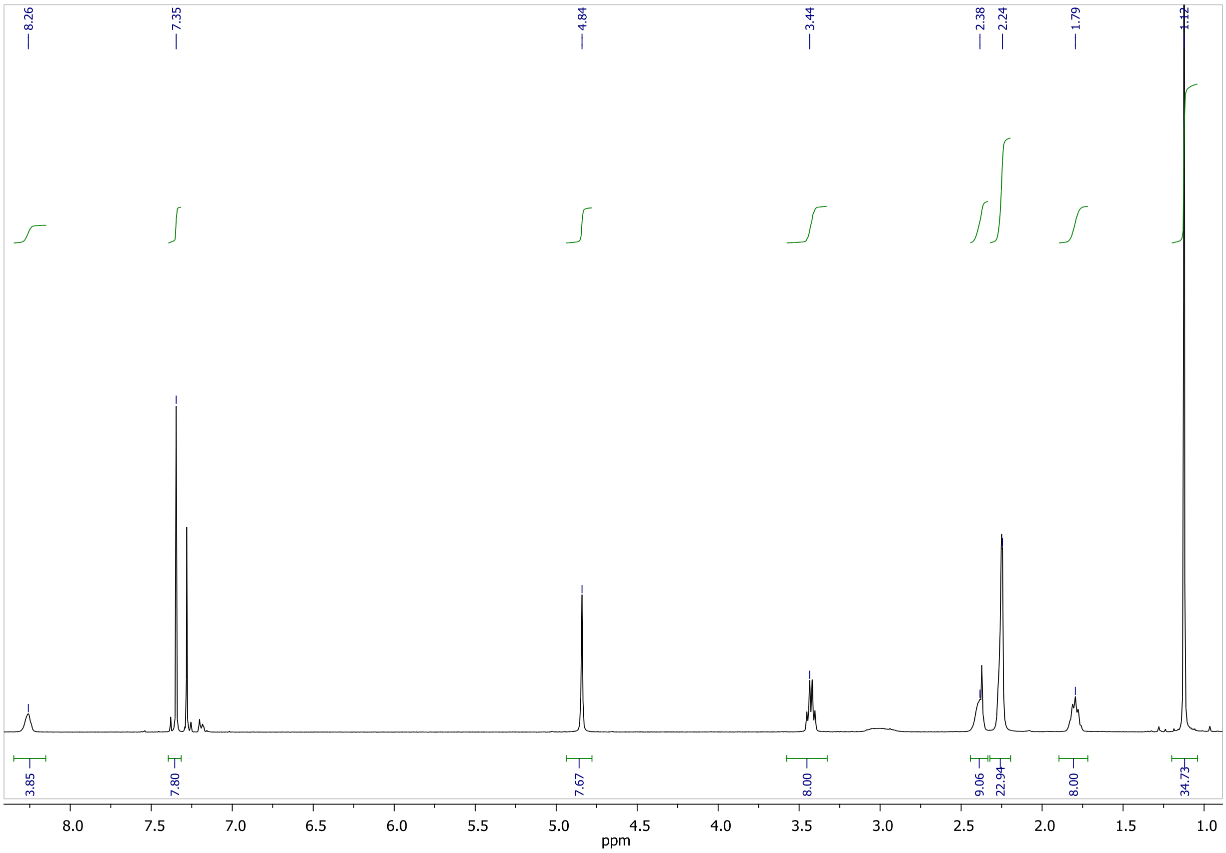

Figure 2S. IR spectrum of macrocycle 1

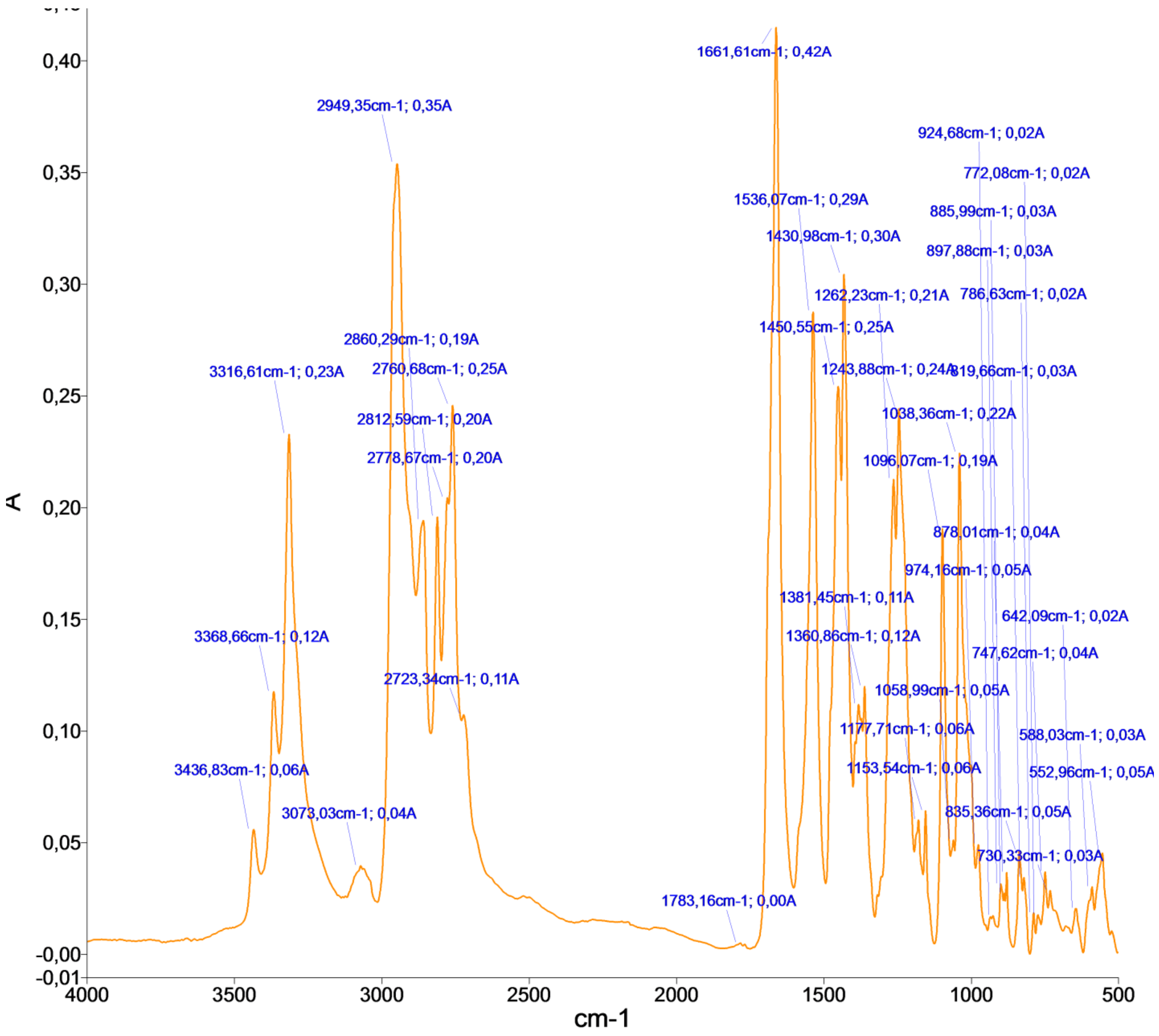

Figure 3S. **NMR ^{1}H spectrum of 5,11,17,23-tetra-*tert*-butyl-25,26,27-[3′-(N-phthalimido)propoxy]-28-[*tert*-butyl(2′-aminoethoxy)carbamate]-2,8,14,20-tetrathiacalix[4]arene (*1,3-alternate*) 3**

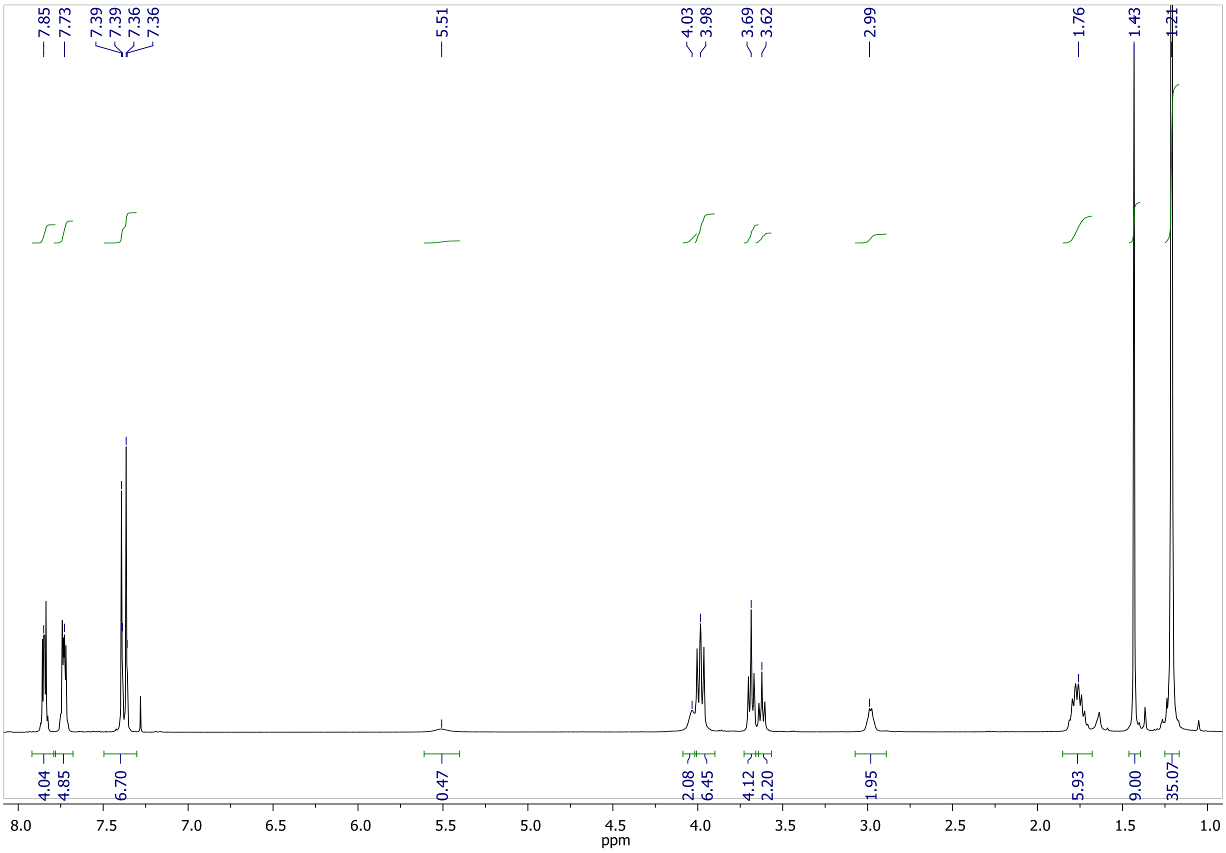

Figure 4S. NMR ^{13}C spectrum of 5,11,17,23-tetra-*tert*-butyl-25,26,27-[3′-(N-phthalimido)propoxy]-28-[*tert*-butyl(2′-aminoethoxy)carbamate]-2,8,14,20-tetrathiacalix[4]arene (*1,3-alternate*) 3

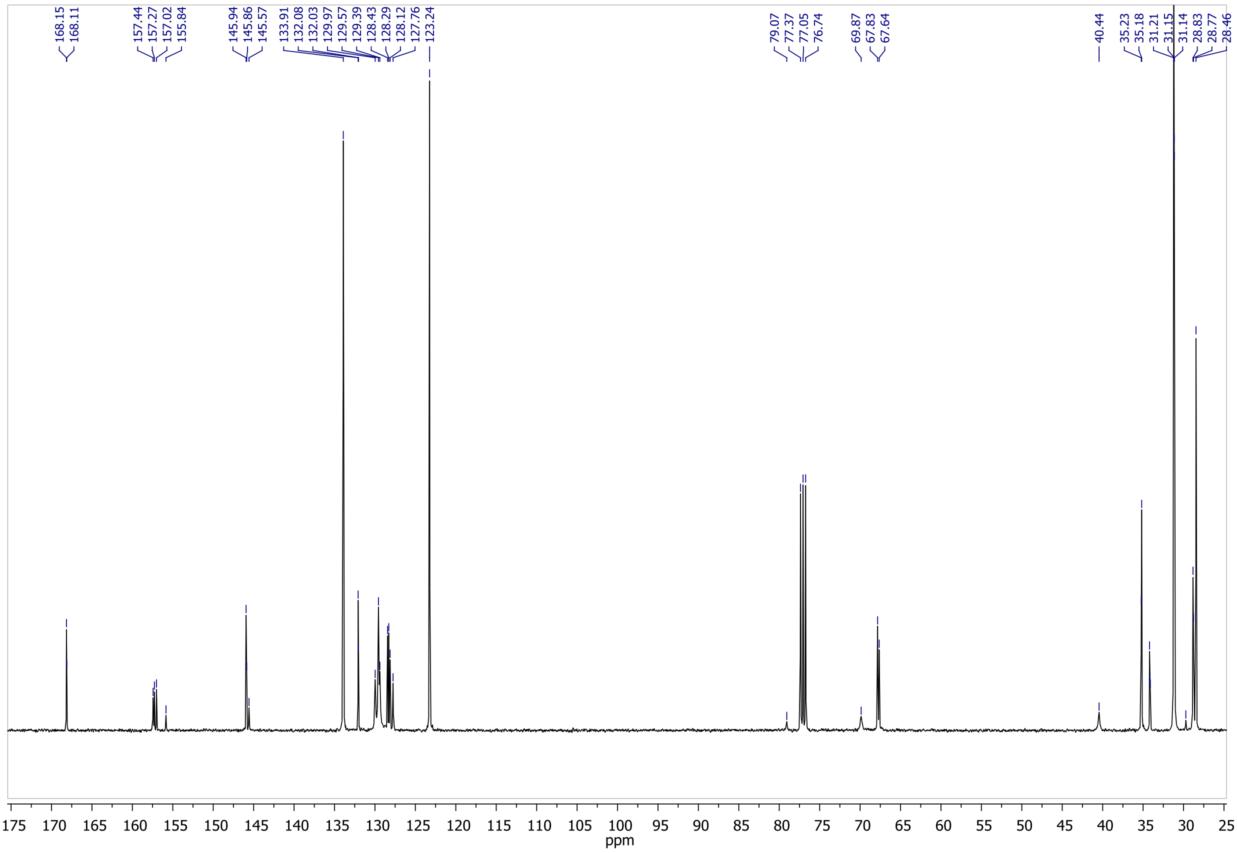

Figure 5S. **NMR ^{1}H-^{1}H NOESY spectrum of 5,11,17,23-tetra-*tert*-butyl-25,26,27-[3′-(N-phthalimido)propoxy]-28-[*tert*-butyl(2′-aminoethoxy)carbamate]-2,8,14,20-tetrathiacalix[4]arene (*1,3-alternate*) 3**

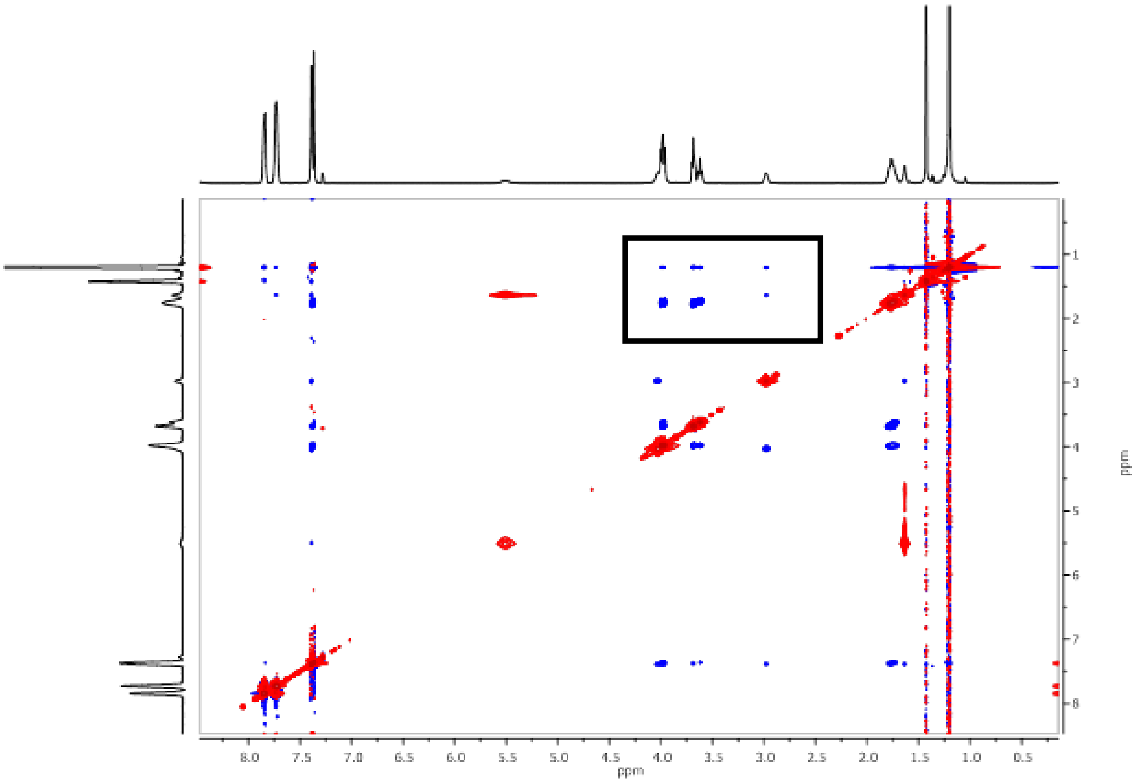

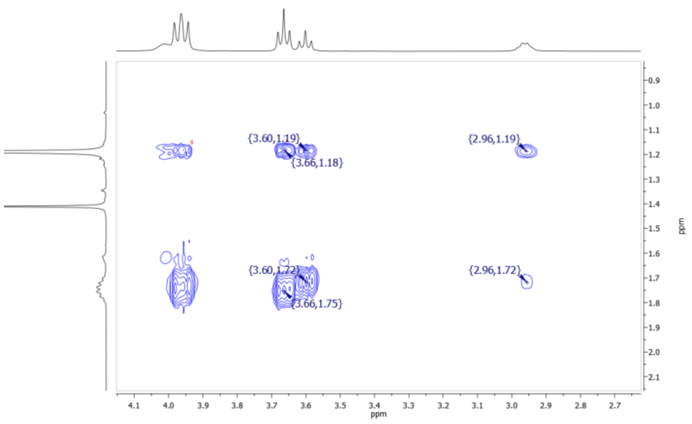

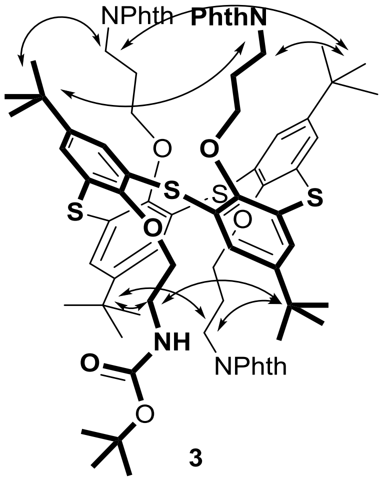

Figure 6S. MALDI TOF spectrum of 5,11,17,23-tetra-*tert*-butyl-25,26,27-[3′-(N-phthalimido)propoxy]-28-[*tert*-butyl(2′-aminoethoxy)carbamate]-2,8,14,20-tetrathiacalix[4]arene (*1,3-alternate*) 3

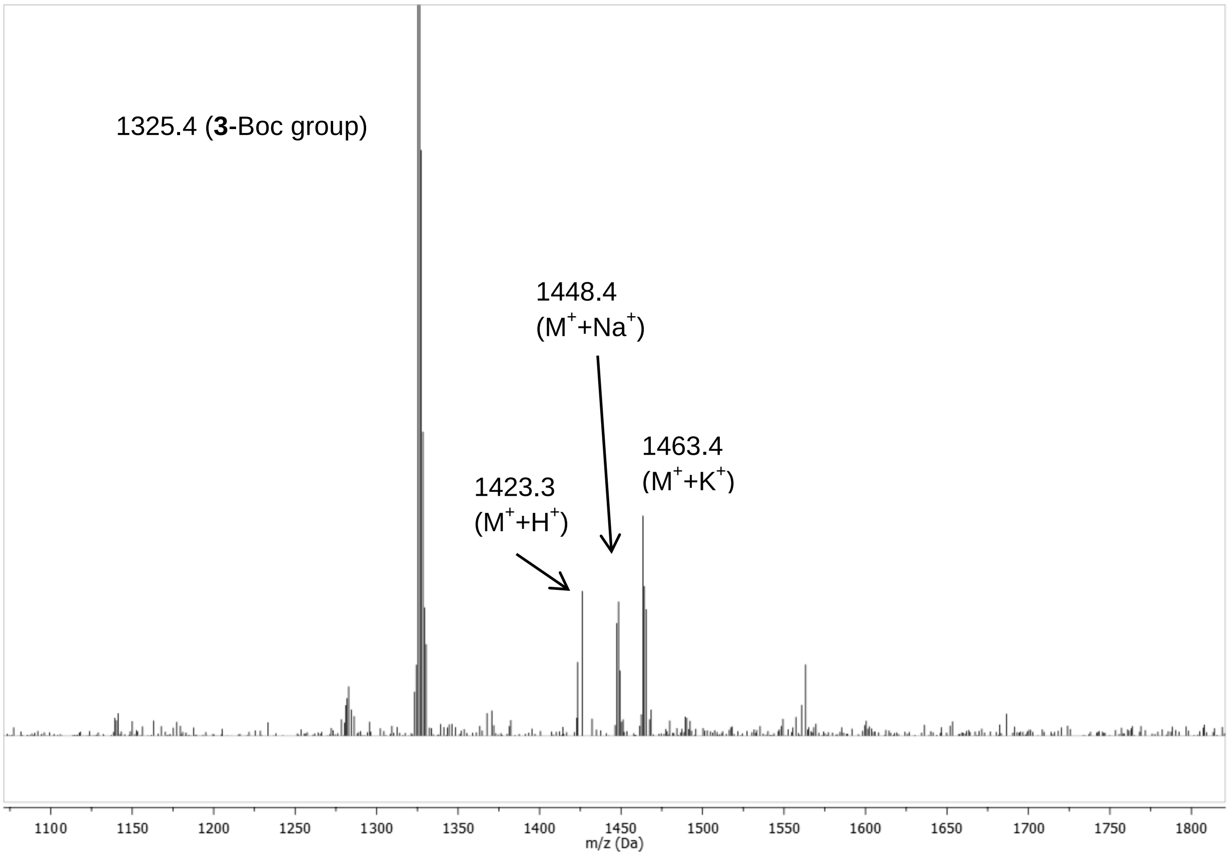

Figure 7S. NMR ^{1}H spectrum of 5,11,17,23-tetra-*tert*-butyl-25,26,27-[3′-(N-phthalimido)propoxy]-28-[2′-aminoethoxy]-2,8,14,20-tetrathiacalix[4]arene (*1,3-alternate*) 4

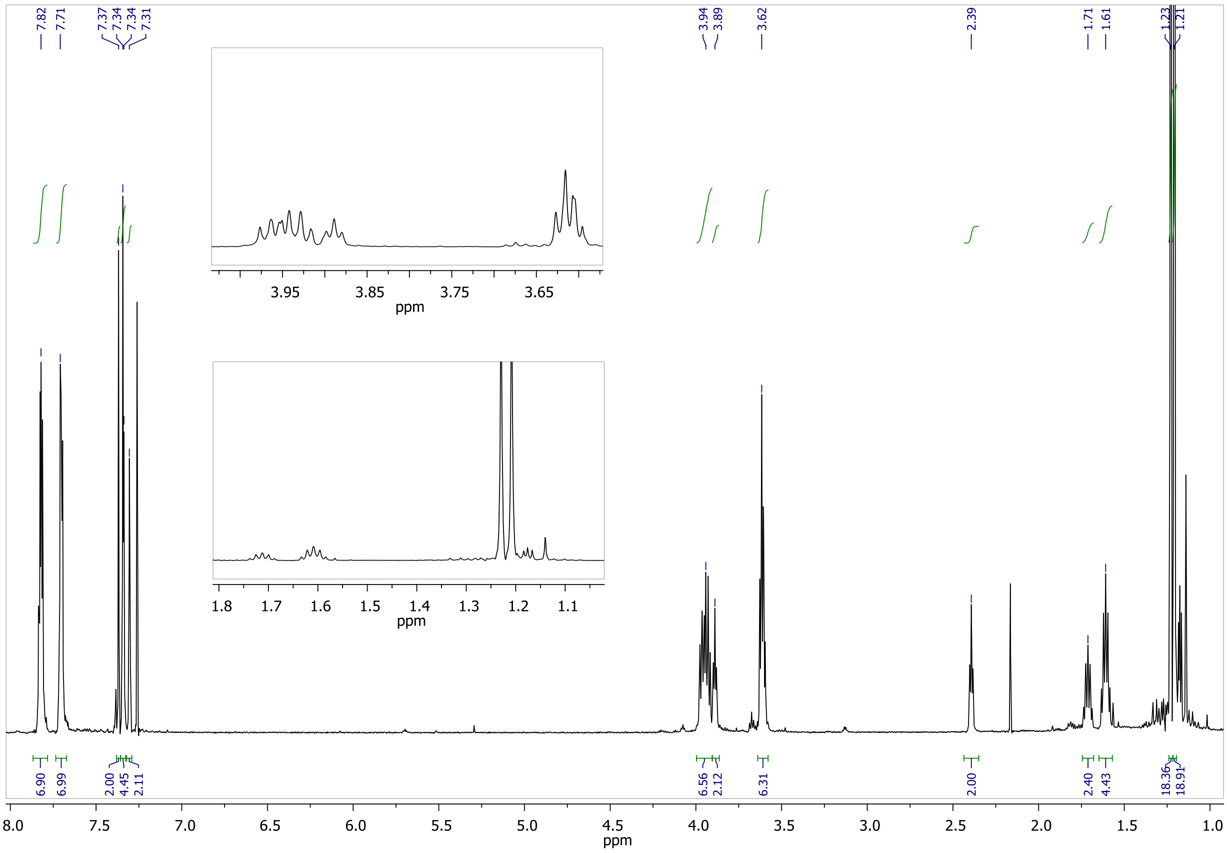

Figure 8S. NMR ^{13}C spectrum of 5,11,17,23-tetra-*tert*-butyl-25,26,27-[3′-(N-phthalimido)propoxy]-28-[2′-aminoethoxy]-2,8,14,20-tetrathiacalix[4]arene 4

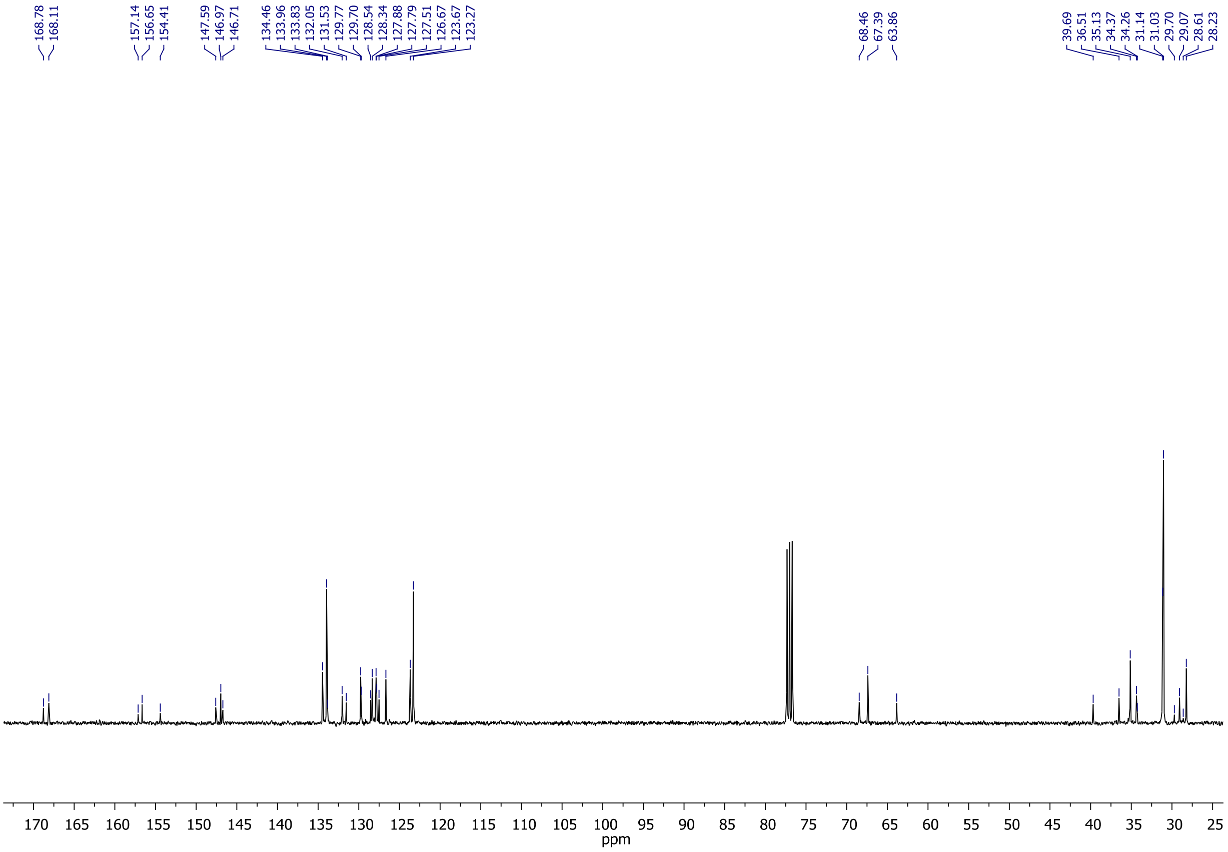

Figure 9S. MALDI-TOF mass spectrum of 5,11,17,23-tetra-*tert*-butyl-25,26,27-[3′-(N-phthalimido)propoxy]-28-[2′-aminoethoxy]-2,8,14,20-tetrathiacalix[4]arene 4

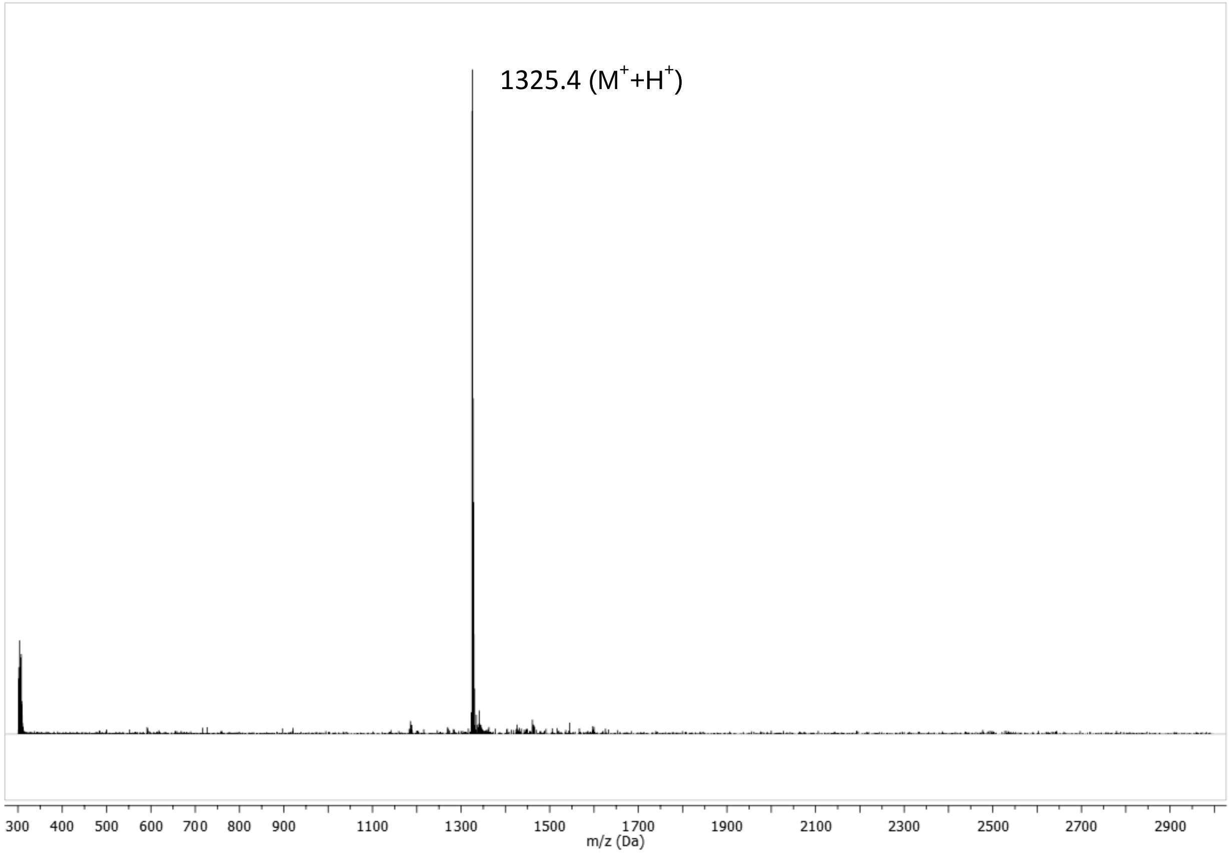

Figure 10S. **NMR ^{1}H spectrum of 5,11,17,23-tetra-*tert*-butyl-25,26,27-[3′-(N-phthalimido)propoxy]-28-[2′-bromoacetamidethoxy]-2,8,14,20-tetrathiacalix[4]arene (*1,3-alternate*) 5**

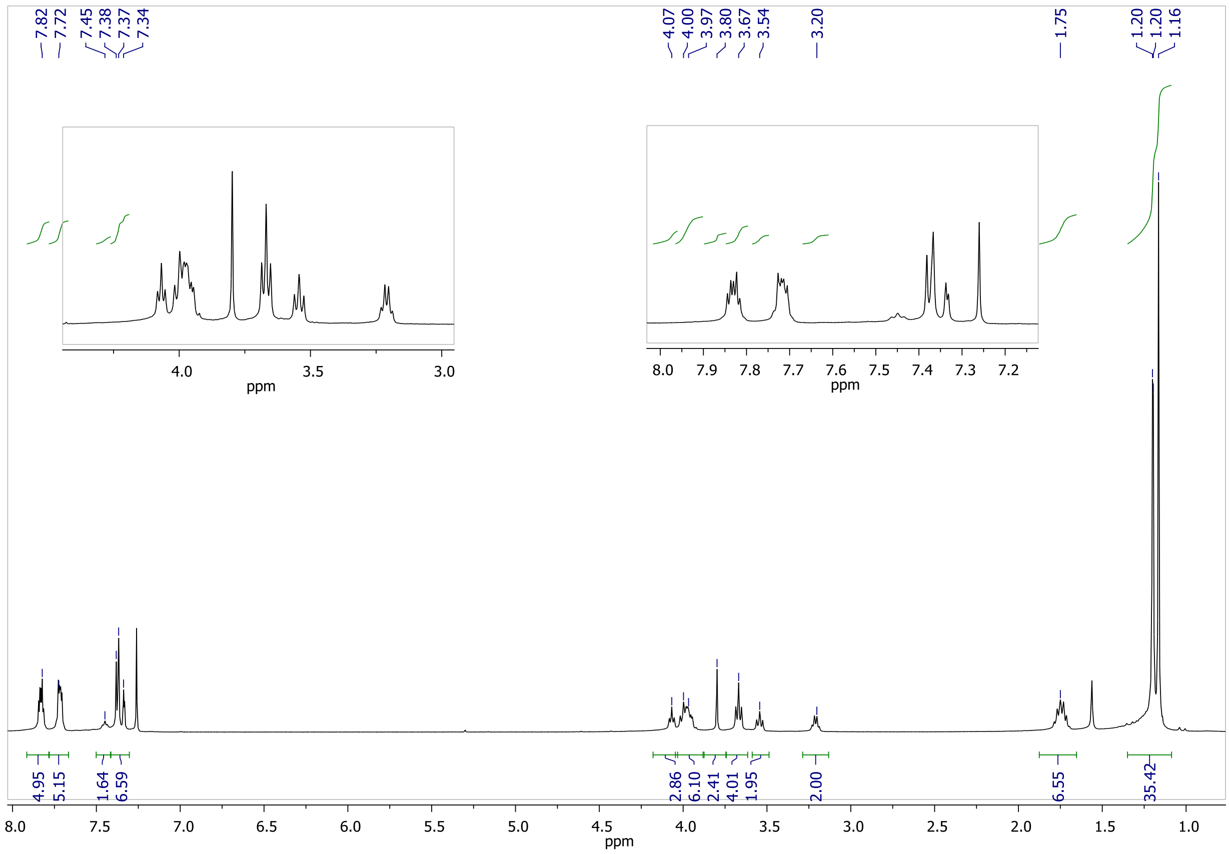

Figure 11S. NMR ¹³C spectrum of 5,11,17,23-tetra-*tert*-butyl-25,26,27-[3′-(N-phthalimido)propoxy]-28-[2′-bromoacetamidethoxy]-2,8,14,20-tetrathiacalix[4]arene (*1,3-alternate*) 5

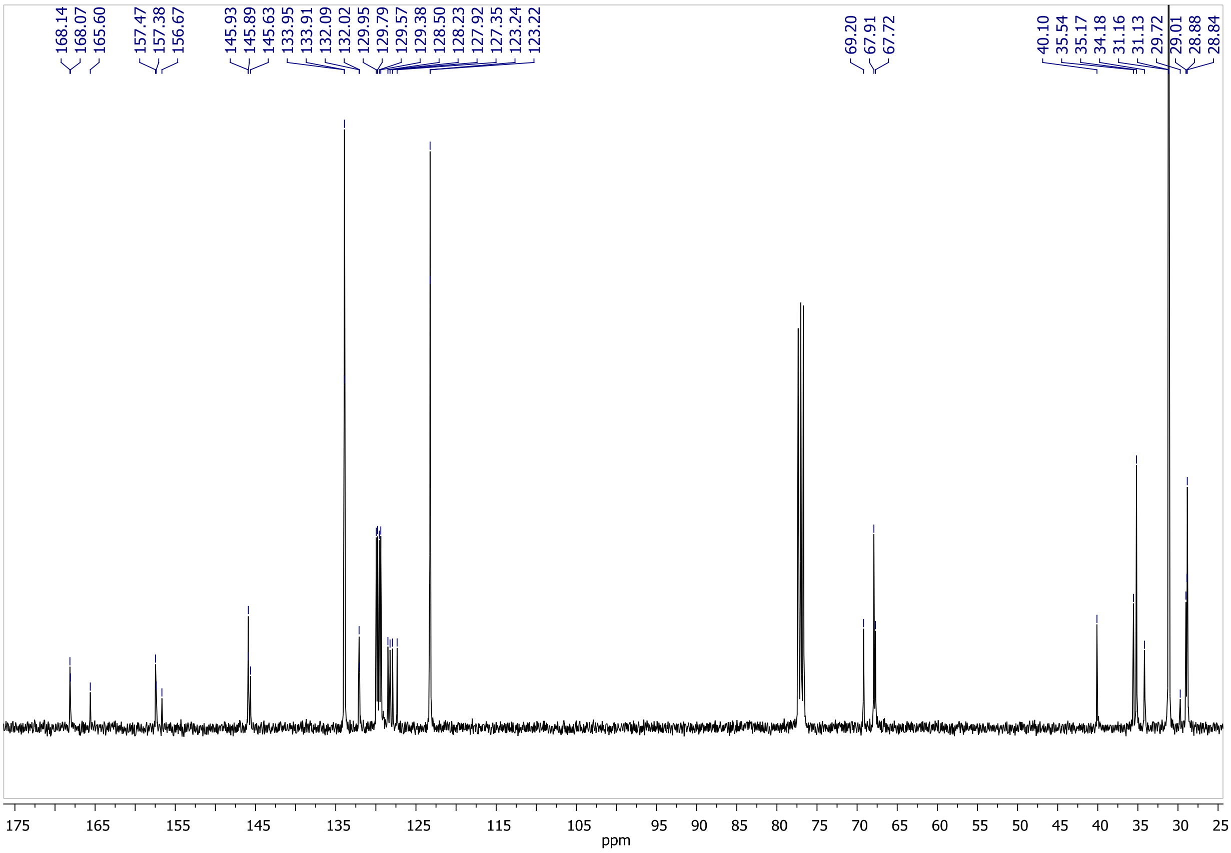

Figure 12S. **NMR ^1H-^1H NOESY spectrum of 5,11,17,23-tetra-*tert*-butyl-25,26,27-[3′-(N-phthalimido)propoxy]-28-[2′-bromoacetamidethoxy]-2,8,14,20-tetrathiacalix[4]arene (*1,3-alternate*) 5**

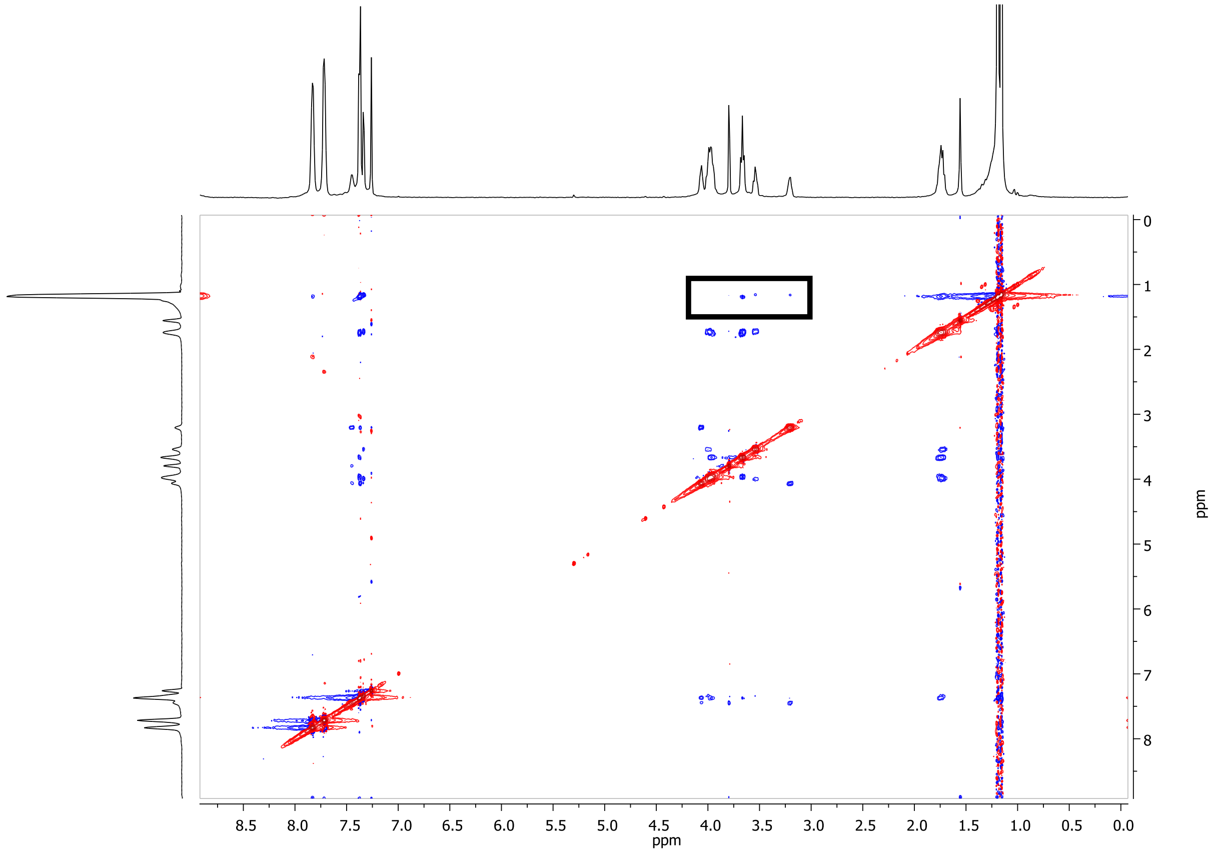

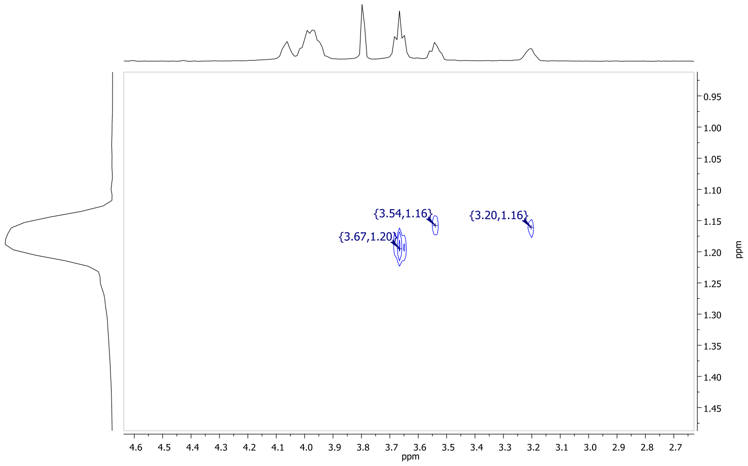

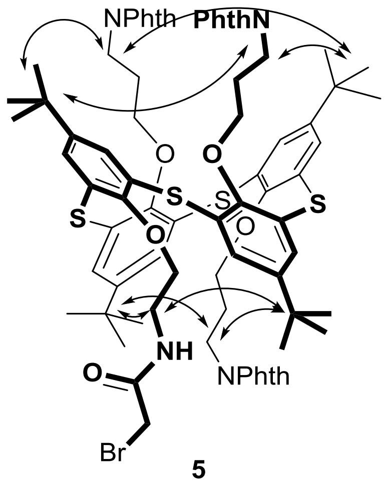

5

Figure 13S. MALDI TOF mass spectrum of 5,11,17,23-tetra-*tert*-butyl-25,26,27-[3′-(N-phthalimido)propoxy]-28-[2′-bromoacetamidethoxy]-2,8,14,20-tetrathiacalix[4]arene (*1,3-alternate*) 5

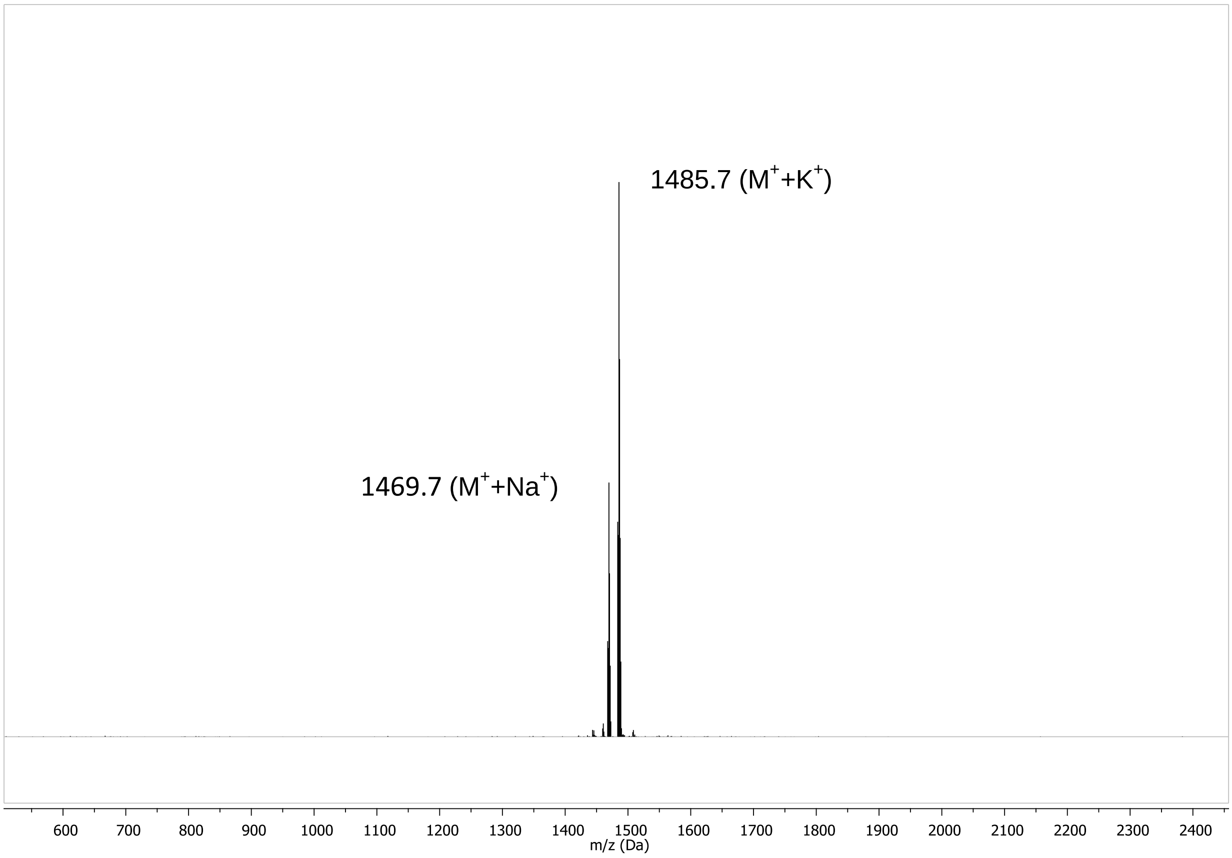

Figure 14S. IR spectrum of 5,11,17,23-tetra-*tert*-butyl-25,26,27-[3′-(N-phthalimido)propoxy]-28-[2′-bromoacetamidethoxy]-2,8,14,20-tetrathiacalix[4]arene (*1,3-alternate*) 5

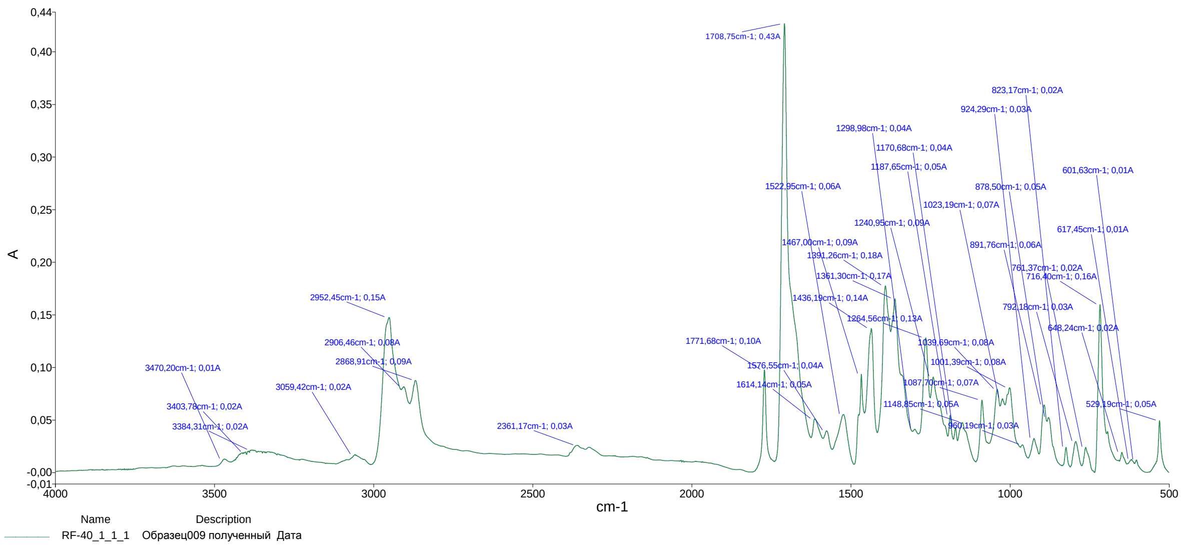

Figure 15S. NMR ^{1}H spectrum of multithiacalix[4]arene 6

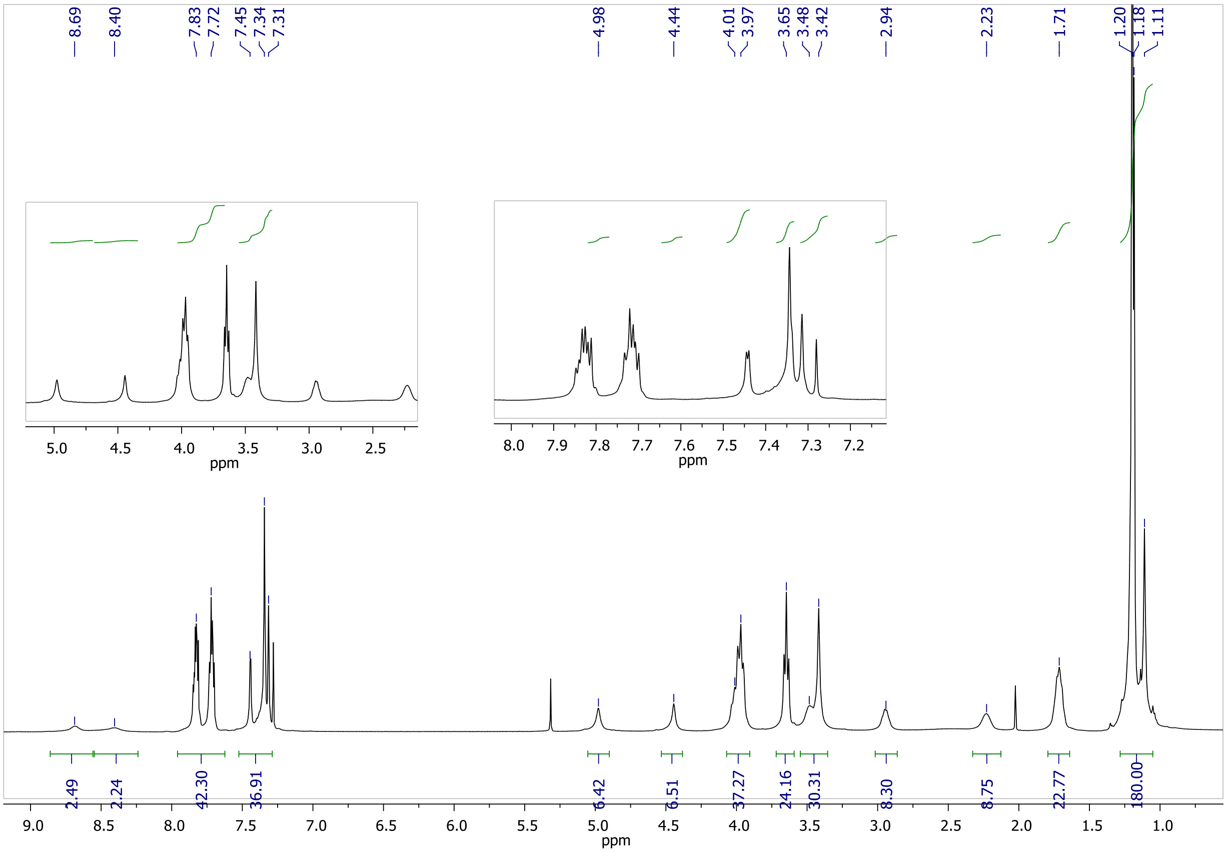

Figure 16S. NMR ^{13}C spectrum of multithiacalix[4]arene 6

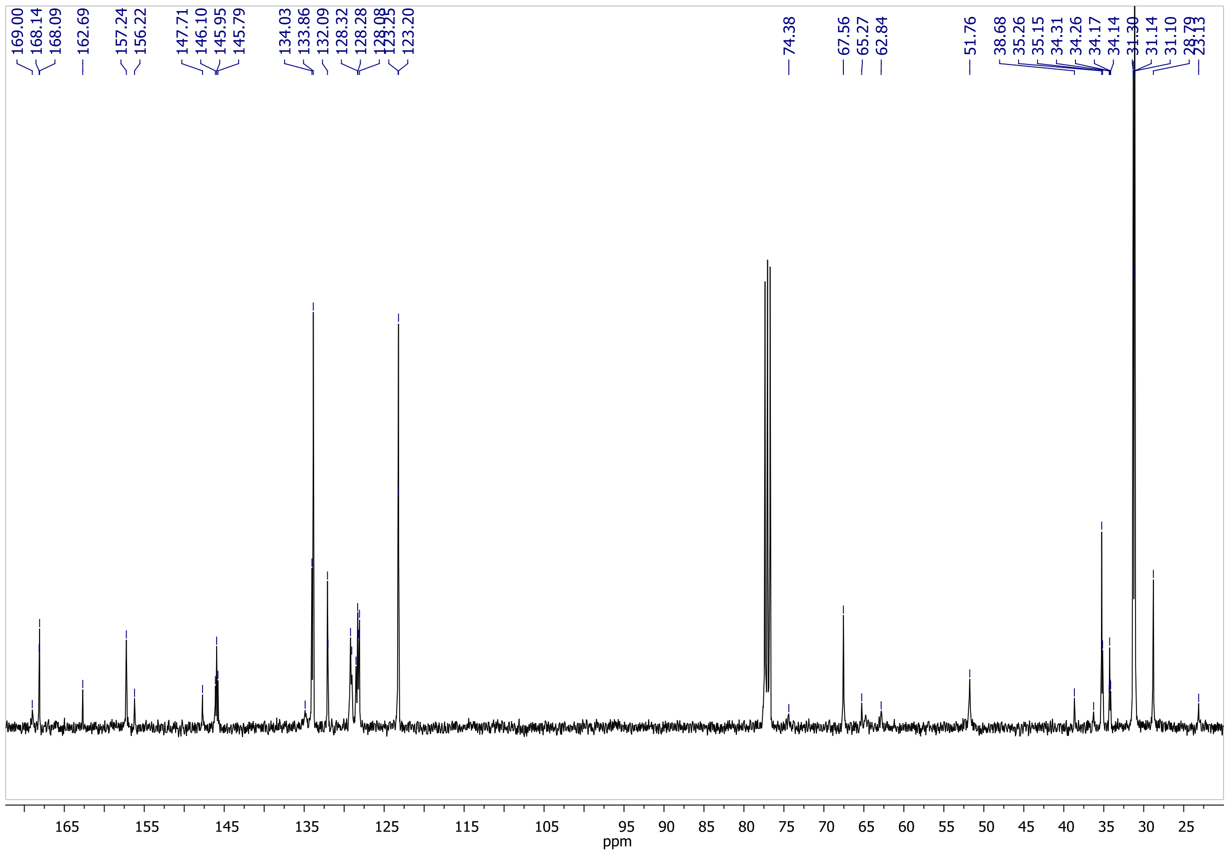

Figure 17S. MALDI TOF mass spectrum of multithiacalix[4]arene 6

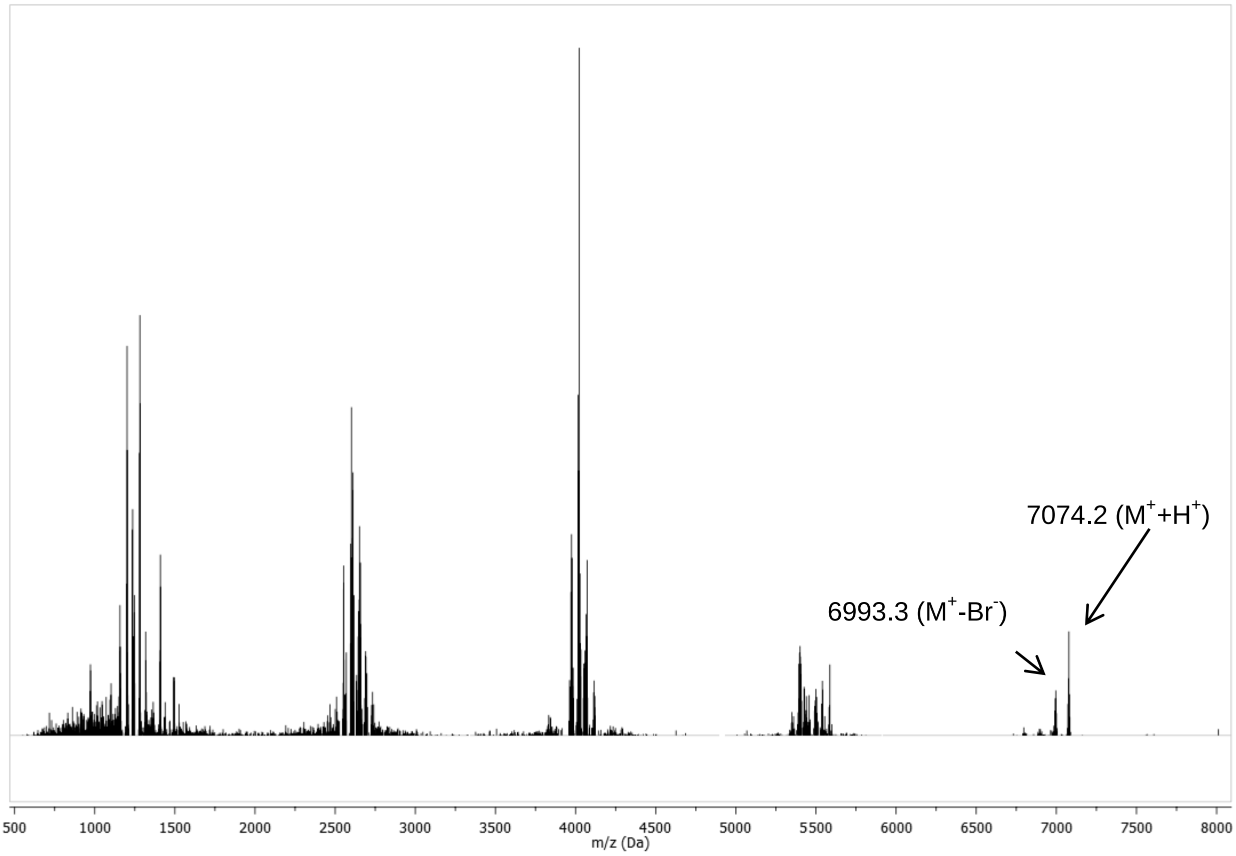

Figure 18S. IR spectrum of multithiacalix[4]arene 6

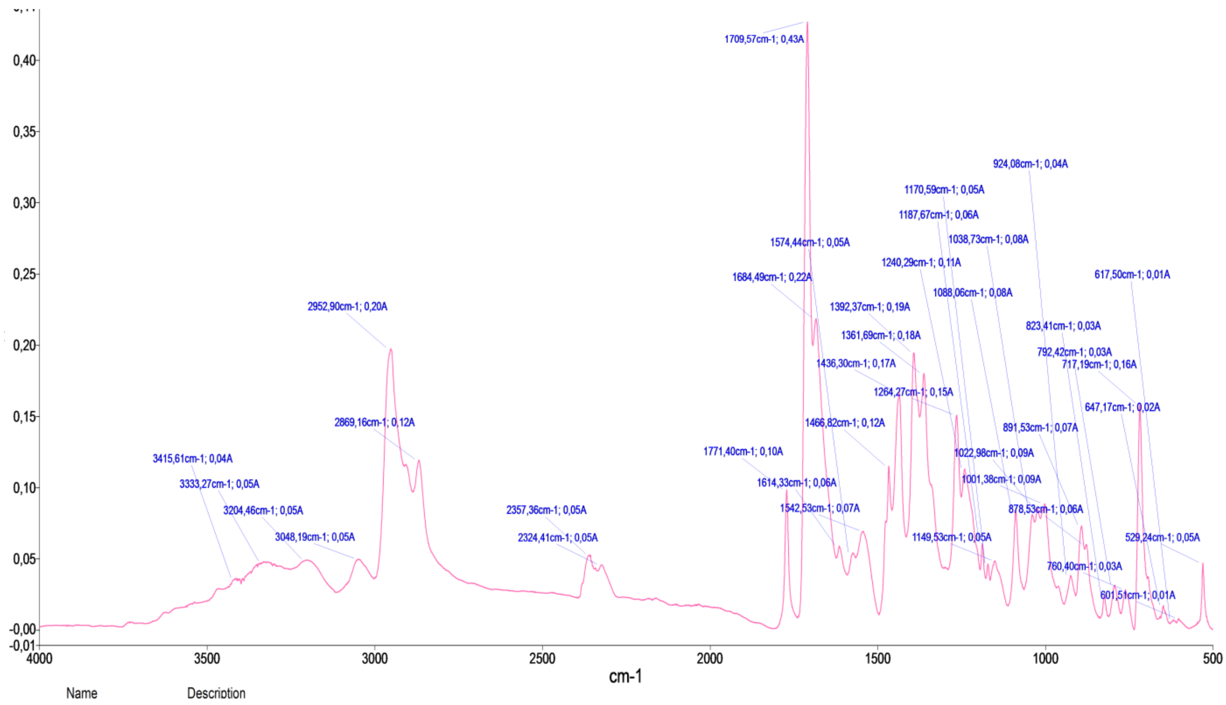

Figure 19S. NMR ^{1}H spectrum of multithiacalix[4]arene 7

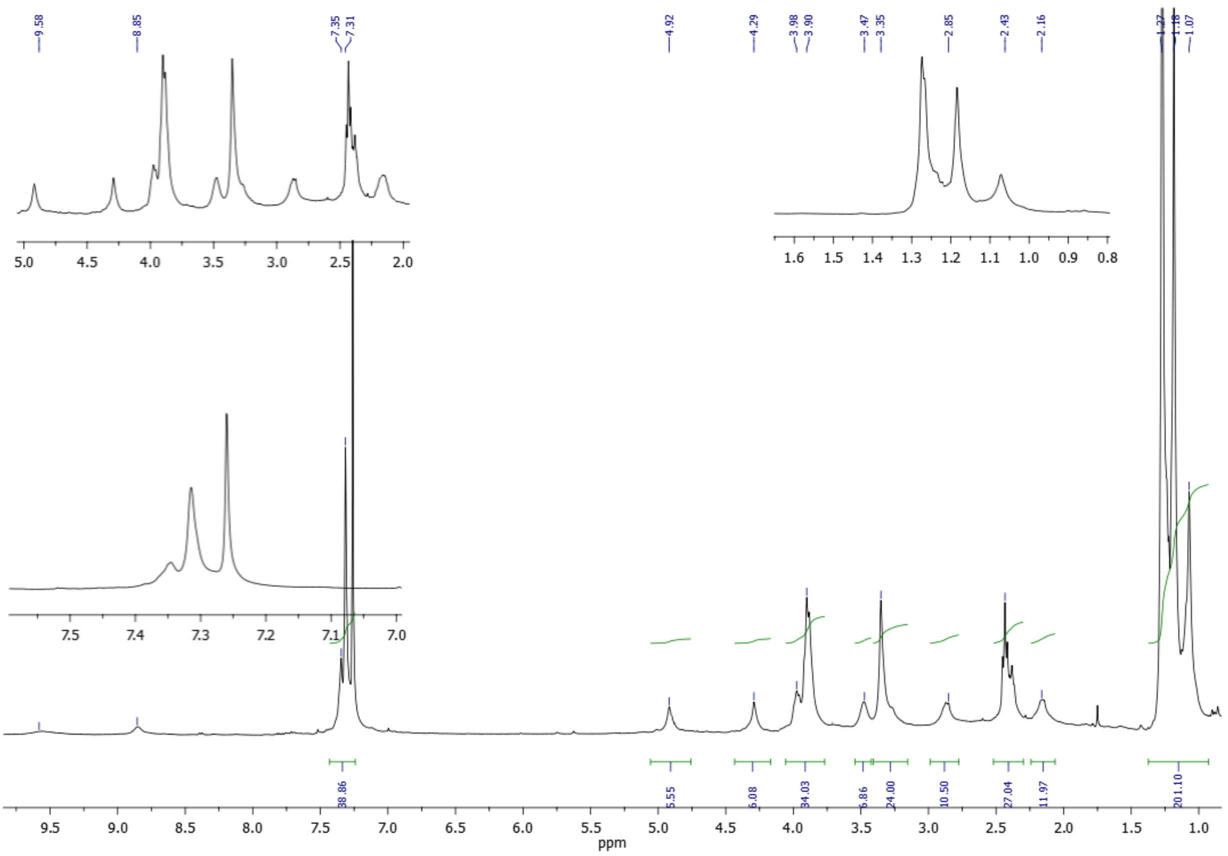

Figure 20S. NMR ^{13}C spectrum of multithiacalix[4]arene 7

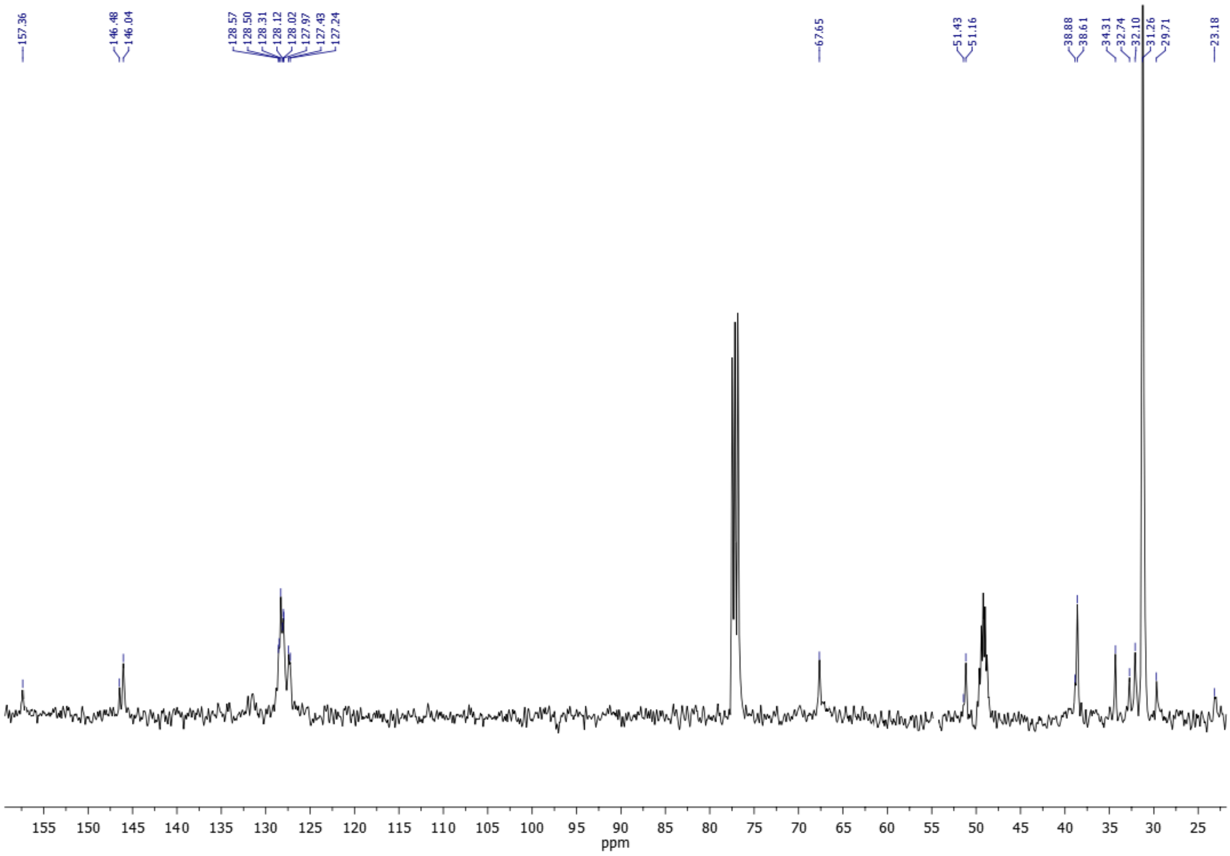

Figure 21S. IR spectrum of multithiacalix[4]arene 7

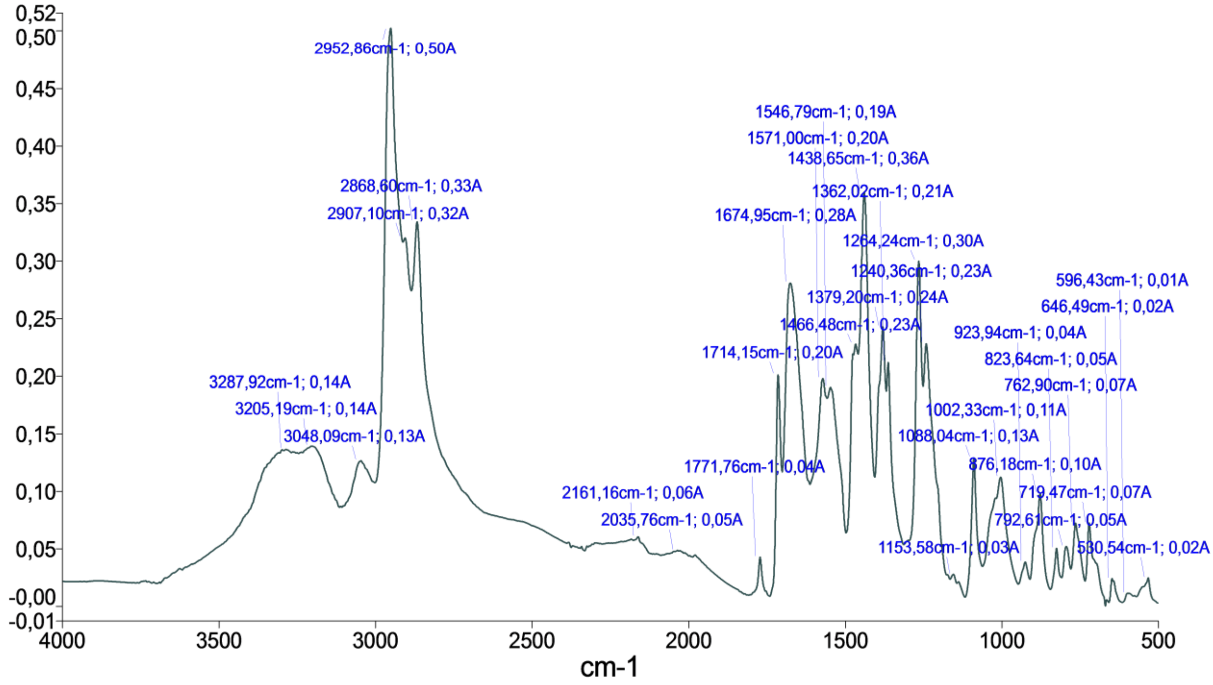

Figure 22S. MALDI TOF mass spectrum of multithiacalix[4]arene 7

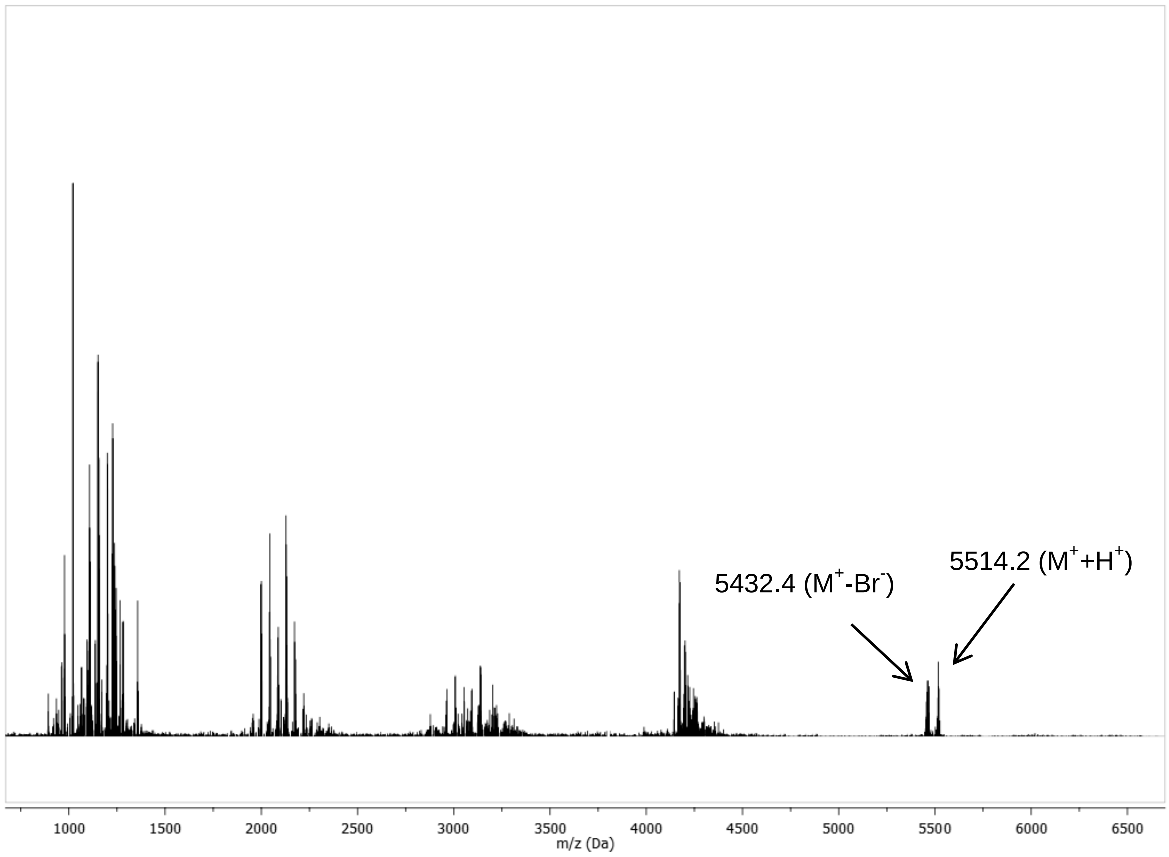

Supplement: Supplementary file 1 [file molecules-23-01117-s001.pdf]
